# New Insights into the Crystal Chemistry of Elpidite, Na_2_Zr[Si_6_O_15_]·3H_2_O and (Na_1+Y_Ca_x_□_1−X−Y_)_Σ=2_Zr[Si_6_O_15_]·(3−X)H_2_O, and Ab Initio Modeling of IR Spectra

**DOI:** 10.3390/ma14092160

**Published:** 2021-04-23

**Authors:** Alexander Bogdanov, Ekaterina Kaneva, Roman Shendrik

**Affiliations:** 1 Vinogradov Institute of Geochemistry, Siberian Branch of the Russian Academy of Sciences, 664033 Irkutsk, Russia; alex.bogdanov2012@gmail.com (A.B.); r.shendrik@gmail.com (R.S.); 2 Department of Subsoil Use, Irkutsk National Research Technical University, 664074 Irkutsk, Russia

**Keywords:** ab initio modeling, IR spectroscopy, elpidite, microporous heterosilicate, ribbon silicate, crystal chemistry

## Abstract

Elpidite belongs to a special group of microporous zirconosilicates, which are of great interest due to their capability to uptake various molecules and ions, e.g., some radioactive species, in their structural voids. The results of a combined electron probe microanalysis and single-crystal X-ray diffraction study of the crystals of elpidite from Burpala (Russia) and Khan-Bogdo (Mongolia) deposits are reported. Some differences in the chemical compositions are observed and substitution at several structural positions within the structure of the compounds are noted. Based on the obtained results, a detailed crystal–chemical characterization of the elpidites under study was carried out. Three different structure models of elpidite were simulated: Na_2_ZrSi_6_O_15_·3H_2_O (related to the structure of Russian elpidite), partly Ca-replaced Na_1.5_Ca_0.25_ZrSi_6_O_15_·2.75H_2_O (close to elpidite from Mongolia), and a hypothetical CaZrSi_6_O_15_·2H_2_O. The vibration spectra of the models were obtained and compared with the experimental one, taken from the literature. The strong influence of water molecule vibrations on the shape of IR spectra of studied structural models of elpidite is discussed in the paper.

## 1. Introduction

Elpidite is an unusual Si-rich hydrous alkaline zirconosilicate, which is characterized by a mixed tetrahedral–octahedral framework, being a representative of microporous heterosilicates. Unlike common zeolites, being aluminosilicates, frameworks of microporous minerals with transition elements are built of both tetrahedral fragments and “strong” cations (Ti, Nb, Zr, Ta, Sn, W, Fe, Mn, Zn, etc.) with a coordination number 6 or 5 [1]. Ion-exchange properties of microporous titano-, niobo- and zirconosilicates are of great interest, taking into account their capability to uptake some radioactive species [2]. In addition, the still unrealized, but promising uses of the interesting properties of these materials, have also been reported [2], such as optical, magnetic, etc.

According to the silicate minerals hierarchy of Day and Hawthorne (2020) [3], elpidite, Na_2_Zr[Si_6_O_15_] 3H_2_O, is a ribbon silicate with a one-dimensional tetrahedral polymerization. The [Si_6_O_15_]^6−^-ribbon in elpidite extends along the *a*-axis. Adjoining ribbons are interconnected by Zr– and Na-polyhedra, forming an open-framework. The silicon–oxygen radical has the designation ^3^T_6_, where T means “tetrahedron”, 3 is the connectivity of the tetrahedron and 6 is the number of such tetrahedra in the geometrical repeat unit [3]. The same type of ribbon (^3^T_6_) can be found in the crystal structures of minerals of the epididymite group, which include epididymite, Na_2_Be_2_[Si_6_O_15_]·H_2_O [4], eudidymite, Na_2_Be_2_[Si_6_O_15_]·H_2_O [4], and yusupovite, Na_2_Zr[Si_6_O_15_]·3H_2_O [5], and related compounds obtained by hydrothermal ion-exchange reactions (Rb_2_Zr[Si_6_O_15_]·*n*H_2_O, K_2_Zr[Si_6_O_15_]·*n*H_2_O [6] and Ag_2_Zr[Si_6_O_15_] *n*H_2_O [7]. It is interesting to note, that in armstrongite, CaZr[Si_6_O_15_]·2H_2_O [8], and dalyite, (K,Na)_2_Zr[Si_6_O_15_] [9], the radical [Si_6_O_15_] does not form a ribbon. Si-complex is represented by corrugated silicate sheets based on the [(4.6.8)_2_(6.8^2^)_1_]_2_ net [10].

The most complete information on the elpidite chemistry and crystal structure research is represented in [11] and summarized in Table S1 of Supplementary Materials.

For elpidite, the calculated framework density (FD—the number of framework knots per 1000 Å^3^ [12]) is 18.2, a value lying in the range (from 14 to 22) found for zeolites and microporous heterosilicates with a framework of tetrahedra and octahedra. The detailed crystal–chemical features of the mineral can help to determine its potential for possible usage in different fields as a material alternative to zeolites.

A number of experiments on the dehydration and thermal stability of elpidite (for example, [6,7,11,13,14,15]) yielded that diffusion within the elpidite structure proceeds via a zigzag track along the c axis. As it is stated in [11,15], at about 100 °C, the crystal structure of elpidite undergoes changes from *Pbcm* to *Cmce* with the doubled *a* parameter. It loses one water molecule (Ow2). The structure becomes anhydrous at about 225 °C. In addition, the *Pbcm* → *Pbca* → *C*1121/*a* → *P*1121/*n* structural transition occurs with increasing pressure from 0.0001 to 4.97 GPa [16]. Finally, a highly hydrated variety of elpidite (sp gr *Pma*2) with the deficiency of Na and presence of H_3_O^+^ has recently been found in the Khibiny complex (Russia) [17].

Infrared (IR) spectroscopy is one of the tools by which the presence of H_2_O molecules is identified. However, the attribution of lines in the spectrum to a particular position of a molecule in the structure is a more difficult task. Structural data allowed for the reliable determination of the cavities’ size in the studied minerals. Such cages are usually occupied by alkaline and alkaline–earth cations and water molecules.

In the present article, based on the crystal–chemical and structural data of natural elpidite from the Burpala (Russia) and Khan-Bogdo (Mongolia) massifs, ab initio calculation is performed to more accurately refine the water positions and study the spectroscopic properties of the compounds. The vibrational spectrum of the tetrahedral–octahedral framework of elpidite is modeled and compared with the experimental one. A detailed study on elpidite from Burpala is presented here for the first time.

## 2. Materials and Methods

### 2.1. Samples Description

The studied elpidite was taken from two complexes of alkaline rocks: the Burpala (Russia) and Khan-Bogdo (Mongolia) massifs. The Burpala massif is located in the North Baikal Highland belonging to the Baikal Alkaline Province. The Khan-Bogdo is one of the world’s largest alkali granite plutons. It is situated in the southern Gobi Desert. Elpidite forms translucent brown crystals with perfect cleavage on {110} plane.

### 2.2. Chemical and Structural Analysis

Electron microprobe analysis (EMPA) was carried out on two single crystals of Burpala elpidite (hereafter ElB) and two single crystals of elpidite from the Khan-Bogdo massif (hereafter ElKhB) samples embedded in epoxy resin, polished and carbon-coated. The same crystals were used for single-crystal X-ray diffraction analysis (SCXRD).

A JEOL JXA-8200 electron microprobe (JEOL, Tokyo, Japan) operating at 15 kV accelerating voltage, 5 nA sample current, ~1 μm spot size, and 40 s counting time was used. The full wavelength-dispersive spectrometry (WDS) mode was employed. The used standards for major, minor, and REE components were: wollastonite (Si), anorthite (Ca, Al), omphacite (Na), F-apatite (F), olivine (Mg), K-feldspar (K), rhodonite (Mn, Zn), fayalite (Fe), celestine (Sr), Zr-jarosite (Zr, Hf), sanbornite (Ba), La-phosphate (La), Ce-phosphate (Ce), Pr-phosphate (Pr), Nd-phosphate (Nd), Sm-phosphate (Sm), Eu-phosphate (Eu), Gd-phosphate (Gd), Dy-phosphate (Dy), Ho-phosphate (Ho), Er-phosphate (Er), Yb-phosphate (Yb), Lu-phosphate (Lu), ilmenite (Ti), Y-phosphate (Y) and pure Cu, V, Cr, Co, Ni, Nb.

For the conversion from X-ray counts to oxide weight percentages (wt.%), a Phi-Rho-Z method was employed as implemented in the Jeol (Tokyo, Japan) suite of the program.

The crystal structure of elpidite samples was studied using a Bruker AXS X8 APEXII automated diffractometer (Bruker, Berlin, Germany) equipped with a four-circle Kappa goniometer, a CCD detector, and monochromatized Mo*Kα* radiation. The operating conditions were 50 kV and 30 mA. The detector-to-crystal working distance was 40 mm. The collection strategy was optimized with the COSMO program in the APEX2 (Version 2014.11-0, Bruker AXS Inc.: Madison, WI, USA) suite package [18]. A combination of several ω and φ rotation sets (0.5° scan width; 10–50 s per frame exposure time) was used for the recording of the entire Ewald sphere (±*h*, ±*k*, ±*l*) up to θ_max_ ~ 40°. The SAINT package was used for the extraction of the reflection intensities and the correction of the Lorenz polarization effect [19]. The SADABS software (Version 2.10, University of Göttingen, Göttingen, Germany) provided for a semi-empirical absorption correction [20], and the XPREP [21] was used for the calculation of the intensity statistics. The structure was refined in the space group *Pbcm* using the CRYSTALS program (Version 12, University of Oxford, Oxford, UK) [22]. The refined parameters were: scale factor, atom positions, anisotropic displacement parameters and Zr, Na (or Na/Ca) cations and Ow anions occupancies. Occupancies for Si and O atoms were constrained to 1. Ionized X-ray scattering curves were used for non-tetrahedral cations and anions, whereas ionized vs. neutral curves were employed for Si and O atoms [23]. Initial fractional coordinates and atom labeling were taken from [24]. The final fully anisotropic structural refinement converged to *R* = 2.12–3.00% (*Rw* = 2.41–3.42%). Summary data about the single crystals, the data-collection parameters and the structural refinements are given in Table 1, whereas final atomic coordinates, site occupancies, equivalent and anisotropic displacement parameters are reported in Tables S2–S9 of Supplementary Materials. Selected interatomic distances and angles are given in Table S10, S11 of Supplementary Materials, respectively.

The CIFs were deposited with the Cambridge Crystallographic Data Centre (CCDC 2069235 and 2069236–elpidite samples from Burpala, CCDC 2069234 and 2069237–Khan-Bogdo elpidite samples) and are also available from the authors.

A statistical analysis of structural data was carried out using the calculation of the characteristics of coordination polyhedra. For this analysis, the calculations of the parameters well described by us earlier in [25] were applied. Geometric data and distortion parameters for elpidite samples are given in Tables S12 and S13 of Supplementary Materials. Bond valence calculations (Tables S14–S17 of Supplementary Materials) were performed using the parameters obtained by Gagné, and Hawthorne (2015) [26].

### 2.3. Calculation Details

The quantum chemical computations were performed on the Density Functional Theory level within the VASP (Vienna Ab initio Simulation Package) code (VASP Software GmbH, Vienna, Austria) [27]. The code utilizes ultrasoft pseudopotentials and plane-wave basis sets. An energy cutoff of 700 eV was chosen for the plane waves. The electrons treated as valent in our calculations were: 4*s*^2^4*p*^6^5*s*^2^4*d*^2^ for Zr, 3*s*^2^3*p*^2^ for Si, 2*s*^2^2*p*^4^ for O, 2*s*^2^2*p*^6^3*s*^1^ for Na, for 3*s*^2^3*p*^6^4*s*^2^ for Ca, 1*s*^1^ for H. The PBEsol (Perdew–Burke–Ernzerhof for solids) [28] exchange–correlation functional was used for both geometry optimizations and finite-displacements calculations. The sampling of the Brillouin zone was performed by using 2 × 1 × 1 meshes of k-points of the Monkhorst–Pack type. The meshes were centered at the gamma point. The calculation procedure is the same as described in detail in [29]. First, we performed accurate geometry optimizations with threshold for forces 0.001 eV/Å. Second, we created sets of displaced structures, ran single-point calculations and collected force constants with Phonopy code [30]. Then, the density functional perturbation theory [31] was used for calculations of Born effective charge tensors, as implemented in the VASP procedures. The Phonopy–Spectroscopy tool (University of Bath, Claverton Down, Bath, UK) [32] was used to model infrared spectra. The Phonopy code is capable of creating displaced structures corresponding to phonon vibrations. Those structures were used to analyze atomic contributions to each phonon mode.

**Figure 1:**
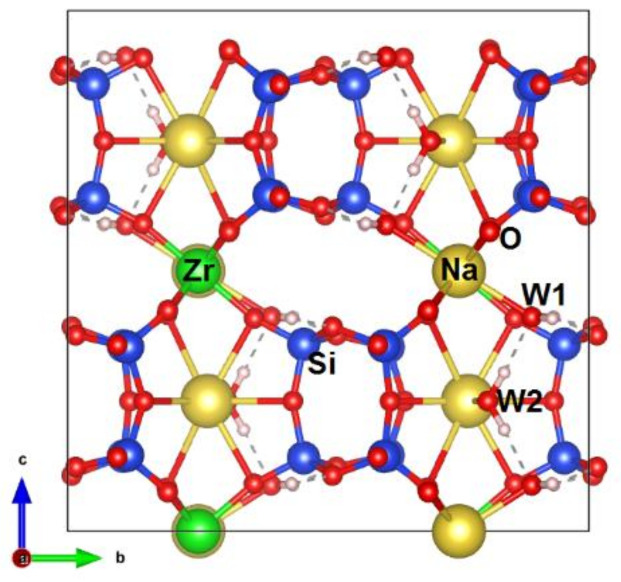
Crystal structure of optimized Na_2_ZrSi_6_O_15_·3H_2_O model.

Figure 1, showing structural details, was prepared using the program VESTA (version 4.3.0, Tsukaba, Kyoto, Japan) [33].

## 3. Results

### 3.1. Chemical Composition and Structure Description

The average compositions (determined over 6–10 spots) are reported in Table 2.

The H_2_O weight percentage and atom proportion in the atoms per formula unit (apfu) were derived from calculation assuming “Total” = 100%. The following crystal–chemical formulas (calculated on the basis of six Si apfu) can be proposed for the studied elpidite samples:

(Na_1.86_K_0.01_)(Zr_0.94_Hf_0.02_REE_0.02_)[Si_6_O_14.79_(OH)_0.21_]·3.10H_2_O and (Na_1.82_K_0.02_Cu_0.01_)(Zr_0.93_Hf_0.02_REE_0.01_)[Si_6_O_14.71_(OH)_0.29_]·3.06H_2_O for elpidite samples from Burpala massif;
(Na_1.21_Ca_0.31_Y_0.03_Fe_0.03_K_0.01_Cu_0.01_Ti_0.01_Mn_0.01_)(Zr_0.94_REE_0.02_Hf_0.01_)[Si_6_O_14.94_(OH)_0.06_]·2.94H_2_O and (Na_1.04_Ca_0.40_Y_0.01_Fe_0.01_K_0.01_)(Zr_0.95_REE_0.01_Hf_0.01_)[Si_6_O_14.79_(OH)_0.21_]·2.79H_2_O for elpidite samples from the Khan-Bogdo massif.

The simplified formulas of studied elpidites are Na_2_Zr[Si_6_O_15_]·3H_2_O and (Na_1+*y*_Ca*_x_*□_1−*x*−*y*_)*_Σ_*_=2_Zr[Si_6_O_15_]·(3−*x*)H_2_O, where □ means “vacancy”, for elpidite from Burpala and Khan-Bogdo, respectively.

Considering that the total negative charge in the ideal formula of elpidite is 30 valence units (v.u.), it can be noted that, in the presence of deficit of the positive charge, the balance is achieved by a decrease in negative charge via O^2−^ → OH^−^ substitutions.

For our specimens, EPMA analyses revealed that the amount of Na is significantly lower and Ca content is higher in Khan-Bogdo elpidite with respect to that from Burpala samples. Grigor’eva et al. (2011) [6] and Zubkova et al. (2019) [7] have reported a markedly lower content of Ca in the samples of elpidite from the Khan-Bogdo massif, (~0.15 vs. ~0.35 atoms per formula unit), whereas Sapozhnikov and Kashaev (1978) [35] described Mongolian elpidite, containing 0.38 Ca^2+^ pfu (Table S1 of Supplementary Materials), consistent with the results of this study. Finally, in comparison with other elpidite compositions (Table 2 and Table S1 of Supplementary Materials), we note that in the studied samples, Zr^4+^ is replaced by Hf^4+^ and REE^3+^, while, for instance, elpidite from the Lovozero massif (Kola Peninsula, Russia) contains trace Hf and Nb [14]. Elpidite from Mt St. Hilaire (Canada) has an increased content of Ti, Mn, Fe and Nb [34]. In addition, recently, a variety of hydrated elpidite from the Khibiny complex (Kola Peninsula, Russia), characterized by a significant deficiency of Na, was described [17].

The heteropolyhedral framework of elpidite consists of a double wollastonite-like silicate chain parallel to the [100] axis. The chains form ribbons linked by ZrO_6_ octahedra. The Na cations and H_2_O molecules are located in extra-framework positions.

In the crystal structure of elpidite, there are: one octahedrally coordinated Zr site, three tetrahedrally coordinated Si sites, two extra-framework Na sites, and two water molecule positions.

According to structure refinement results, the Zr site occupancies are ~1.04 and ~1.02 for elpidite from the Burpala and Khan-Bogdo massifs, respectively, pointing out a presence of elements with higher electron density with respect to Zr. Electron microprobe investigation revealed the presence of a minor amount of Hf^4+^ and rare earth elements in this site. The average values of Zr–O distances are 2.074(3)–2.080(2) Å and 2.082(2) for elpidite from the Burpala and Khan-Bogdo massifs, respectively. The O–Zr–O angles vary from 86.37(9)° to 91.52(9)° for elpidite from Burpala and 86.07(6)° to 91.98(6)° from Khan-Bogdo elpidite, with an average of 90° for both samples (Table S11 of Supplementary Materials). The similar distances, angles and Zr-octahedra volumes (~11.90–11.97 Å^3^, see Tables S10–S12 of Supplementary Materials) indicate similar site content for both specimens.

Each Zr-octahedron is connected to Si1-, Si2- and Si3-tetrahedra. The Si–O distances range from 1.58–1.59 (to apical O atom) to 1.61–1.64 Å (to bridging O atoms) for elpidite from the Burpala massif and from 1.57–1.59 (to apical O atom) to 1.61–1.64 Å (to bridging O atoms). The non-bridging Si–O bond distances are shorter than bridging Si–O ones. In addition, the Si-tetrahedra are slightly distorted in both elpidite structures; distortion parameters are close but not fully in accord (Table S13 of Supplementary Materials).

Sodium ions are located in two extra-framework cavities (Figure 1). One of the Na atoms (Na1) is coordinated by seven oxygens and a water molecule (Ow2—oxygen atom of this water molecule). The Na1 polyhedron volumes are ~29.8–30.1 and ~30.1 Å^3^ for elpidite from Burpala and Khan-Bogdo, respectively. In elpidite from the Burpala massif, the Na1 site is fully occupied, while the same site of Mongolian elpidite is only partially occupied. Cations in the Na2 site are octahedrally coordinated and surrounded by four oxygens and two symmetrically equivalent water molecules (Ow1—oxygen atom of H_2_O). Na2 is completely occupied by Na^+^ in elpidite from the Burpala massif, whereas in the structure of elpidite from the Khan-Bogdo massif, this site is occupied by ~0.33–0.47 Ca^2+^ and ~0.40–0.61 Na^+^ (Tables S2, S4, S6 and S8 of Supplementary Materials). It must be noted that all the Ca content is concentrated in the Na2 site. Its mean atomic number is ~13.3–13.9 e^−^. The Na2 polyhedron volume is 18.7 and 18.4–18.5 Å^3^ for Burpala and Khan-Bogdo elpidite specimens, respectively. The values are significantly smaller than those of Na1 sites ~29.8–30.1 Å^3^.

In the Burpala elpidite, both Ow sites are fully occupied, whereas Ow1 and Ow2 sites of elpidite from the Khan-Bogdo massif have occupancies of ~0.95 and 0.63–0.75, respectively (Tables S2, S4, S6 and S8 of Supplementary Materials). Note also that it was reported that hydronium cations might substitute water molecules in the Ow2 site [17].

### 3.2. IR Spectra Simulation

Despite the fact that numerous IR spectra were obtained earlier for elpidites from different deposits [6,7,14,34,36] (see Table S19 of Supplementary Materials) the attribution of spectral lines is still an ambiguous task. For this purpose, quantum chemical calculations were used for the first time to study the spectroscopic features of elpidites with different chemical compositions.

Three different models with elpidite structure were simulated: Na_2_ZrSi_6_O_15_·3H_2_O, partly Ca-replaced Na_1.5_Ca_0.25_ZrSi6O_15_·2.75H_2_O, and a hypothetical CaZrSi_6_O_15_·2H_2_O. In the Na_1.5_Ca_0.25_ZrSi6O_15_·2.75H_2_O, one H_2_O molecule (Ow2-4 in Table S18 of Supplementary Materials) was removed, and two Na atoms nearest to this vacant H_2_O position were replaced by one Ca. In the CaZrSi_6_O_15_·2H_2_O, four H_2_O molecules (Ow2-1, Ow2-2, Ow2-3, and Ow2-4 in Table S18 of Supplementary Materials) were removed, and all Na atoms were replaced by Ca.

For the first two structures, their lattice vectors were kept fixed during calculations at experimental values for ElB-1. The third, CaZrSi_6_O_15_·2H_2_O, was modeled within the cell of ElKhB-1. Three different models with elpidite structure were simulated: Na_2_ZrSi_6_O_15_·3H_2_O, partly Ca-replaced Na_1.5_Ca_0.25_ZrSi6O_15_·2.75H_2_O, and a hypothetical CaZrSi_6_O_15_·2H_2_O. In the Na_1.5_Ca_0.25_ZrSi6O_15_·2.75H_2_O, one H_2_O molecule (Ow2-4 in Table S18 of Supplementary Materials) was removed, and two Na atoms nearest to this vacant H_2_O position were replaced by one Ca. In the CaZrSi_6_O_15_·2H_2_O, four H_2_O molecules (Ow2-1, Ow2-2, Ow2-3, and Ow2-4 in Table S18 of Supplementary Materials) were removed, and all Na atoms were replaced by Ca.

An important observation is that bond-valence considerations do not preclude any further substitution of the Na^+^ atom by Ca^2+^ in the Na2 position. Thus, the elpidite minerals from the Burpala and Khan-Bogdo massifs could be end members and intermediate members, respectively, of Na_2_Zr[Si_6_O_15_]·3H_2_O–CaZr[Si_6_O_15_]·2H_2_O series. The second end member has not yet been found in nature or synthesized. This structural formula corresponds to armstrongite [8], but this mineral crystallizes in a space group *C*2/*m* and has a completely different type of structure (as it was noted in the Introduction chapter). However, for the hypothetical structural model, the IR spectrum was also calculated to better understand the specific vibrational features of the phases under study.

The calculated values of the peak positions and their assignments are given in Table 3 and expanded in Tables S20 and S22 of Supplementary Materials.

## 4. Discussion

The complementary measure of the strain of the whole crystal structure is expressed in the global instability index (GII), defined by Salinas-Sanchez et al. (1992) [37]. As seen (Table 4), Zr, Na, Si, and O are in the medium range, taking into account that GII < 5% suggest little or no strain is present, and values > 20% indicate unstable structures [38]. Elpidite from Khan-Bogdo (sample ElKhB-2) shows a significantly increased index for Na and a low value of GII (%) Zr (12.79 and 0.40%, respectively). The incorporation of calcium leads to local structural strain, indicating higher instability. Assuming GII total values, both samples’ structures (Burpala and Khan-Bogdo minerals) can be considered stable (GIIs total range from 8.65 to 11.19%). However, the crystal structure of the Burpala elpidite is more relaxed.

Taking into account the observed cation and anion distribution, the following isomorphous substitution schemes can be suggested for elpidite from the Burpala massif: Na^+^ + O^2−^ ↔ □ + OH^−^; Zr^4+^ ↔ Hf^4+^; Zr^4+^ + O^2−^ ↔ REE^3+^ + OH^−^.

Pekov et al. (2009) [39] reported that O^2−^ → (OH)^−^ replacements control the charge balance in cation-deficient members of the zirsinalite–lovozerite group, representative of microporous minerals with heteropolyhedral framework. According to the calculation of the local valence balance (Tables S14–S17 of Supplementary Materials), the common vertices of the SiO_4_ tetrahedra and ZrO_6_ octahedra are presumed to be partially occupied by the OH^−^ groups. In fact, O3, O6 and O9 anions are somewhat undersaturated, with bond valence sums of ~1.89–1.94 v.u. (Tables S14–S17 of Supplementary Materials), that will be strongly stressed by the lacking of Na^+^ (i.e., in a possible leaching process of Na) and for substitutions of Zr^4+^ for REE^3+^. Accordingly, they are potential acceptors of hydrogen bonds, that can confirm the occurrence of (OH)^−^ groups. We have suggested a similar substitution scheme for vlasovite from the Burpala massif [40].

For elpidite from the Khan-Bogdo massif, the isomorphous substitution schemes are resulted to be more complex with respect to the Burpala mineral: Zr^4+^ + O^2−^ ↔ REE^3+^ + OH^−^, Zr^4+^ ↔ Hf^4+^; 2Na^+^ + H_2_O ↔ Ca + 2□; Na^+^ + O^2−^ ↔ □ + OH^−^. These substitution processes demonstrate that the ion-exchange process in elpidite from the Khan-Bogdo massif has a selective nature. Therefore, one type of ion exchange in elpidite realizes in the Na2 site via the substitution by Ca ions (having ionic radius similar to Na) and the removal of one water molecule (Ow2). Another one concerns the ousting of Na^+^ cation from Na1 position, located in a more voluminous coordination polyhedron, and the occurrence of vacancy balanced by OH^−^ → O^2−^ substitution. Na → Ca isomorphism in the structure seems to involve three structural positions: two sodium and a water site.

For an accurate structural characterization, the distortion characteristics of the studied phases are provided. The tetrahedra angle variance (TAV, [41]) of the Si2 site, whose vertices are common as with Si1 and Si3, as well as with Si2, is lower than the ones for Si1 and Si3, whose vertices are common only with symmetrically equivalent tetrahedra and with the Si2. At the same time, the parameters of ELD (edge length distortion, [42]) and BLD (bond length distortion, [42]) for Si2 are slightly lower and slightly higher, respectively, in comparison with Si1 and Si3. Figures S1 and S2 of Supplementary Materials show a relationship between these distortion parameters listed in Table S13 of Supplementary Materials for the tetrahedral sites of the studied minerals.

The comparison of distortion parameters for Zr and Na octahedra shows significantly larger differences than those in the Si-tetrahedra. The Vp (volume of the coordination polyhedron) and Vs (volume of the sphere fitted to the positions of ligands) for the Na2 site in elpidite from Burpala are larger than the Vp and Vs for Na2 in Mongolian samples. ECCv (volume eccentricity) calculated by IVTON (University of Copenhagen, Copenhagen, Denmark) [43] varies moderately, amounting to ~0.01 for Zr octahedron, and reaching ~0.15 for Na1 polyhedron. Figure S3 of Supplementary Materials shows a good correlation between the volume sphericity (SPHv) vs. polyhedral volume (Vp). The Vp and Vs of Na1 are much larger than Na2 and Zr (Table S12 and Figure S3 of Supplementary Materials).

The structural geometric parameters of the optimized models are comparable to calculations performed for the experimentally obtained crystal structure of elpidite from the Burpala and Khan-Bogdo massifs (see Table S21 of Supplementary Materials). Comparing the simulated Na_2_ZrSi_6_O_15_·3H_2_O model with the elpidite from the Burpala, only a few insignificant differences are noted: (1) the values of the average bond length for Si atoms in the simulated model are slightly higher than those in the experimental one: 1.624–1.627 Å vs. 1.611–1.618 Å; (2) the values for the volumes of tetrahedra obey the same tendency: 2.187–2.200 Å^3^ vs. 2.139–2.167 Å^3^ for simulated and experimental models, respectively; (3) for Na1 polyhedron: average bond length = 2.633 and 2.645–2.652 Å and polyhedra volume = 29.195 and 29.785–29.999 Å^3^ for simulated and experimental models, respectively. Regarding the comparison of the Na_1.5_Ca_0.25_ZrSi_6_O_15_·2.75H_2_O model with the Ca-enriched elpidite from Khan-Bogdo, the following small differences are noted: (1) the TAV and OAV (octahedra angle variance) values for tetrahedra and octahedra are slightly increased in the simulated model (Table S21 of Supplementary Materials); (2) the values of average interatomic distances and volumes of tetrahedra are also higher for the theoretical Na_1.5_Ca_0.25_ZrSi_6_O_15_·2.75H_2_O model (<Si-O> = 1.623–1.629 Å vs. 1.611–1.614 Å and V_tetrahedra_ = 2.186–2.211 Å^3^ vs. 2.137–2.155 Å^3^ for simulated and experimental models, respectively; (3) Na1 polyhedron in the simulated model has values of the average bond length slightly lower than that in the experimental model (2.558–2.597 Å vs. 2.653–2.654 Å); (4) the same applies accordingly to the values of the volumes of Na1 polyhedron. In general, the structural and geometric features of the simulated models and the experimental ones are very close to each other. The structural parameters calculated for the hypothetical CaZrSi_6_O_15_·2H_2_O model are presented in Table S22 of Supplementary Materials. Analysis of the table shows that the calculated geometric parameters are not knocked out of the ranges of values obtained for the previously discussed simulated and experimental models.

Thus, since the optimized models have no structural deviations, we assume it is possible to compare the IR spectra obtained by ab initio modeling with the experimental IR spectra of elpidites.

**Figure 2:**
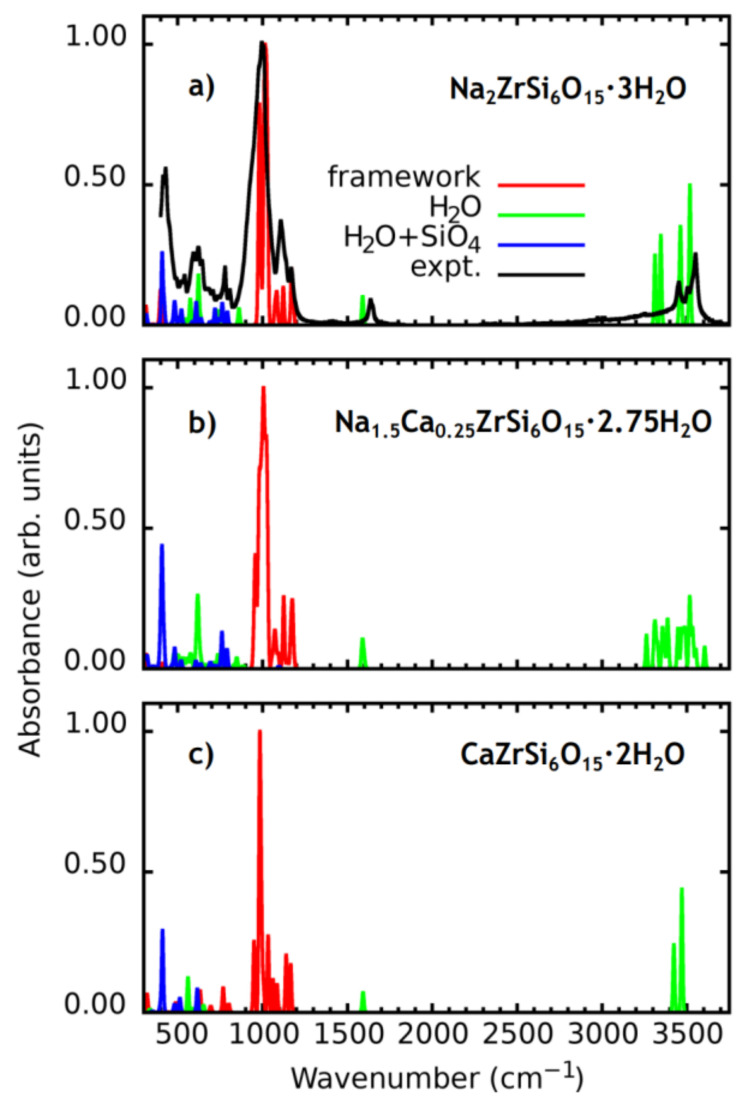
Calculated infrared spectra of (**a**) Na_2_ZrSi_6_O_15_·3H_2_O, (**b**) Na_1.5_Ca_0.25_ZrSi_6_O_15_·2.75H_2_O and (**c**) CaZrSi_6_O_15_·2H_2_O structure models. The H_2_O + SiO_4_ key states for SiO_4_ bending vibrations perturbed by H_2_O. Near 1000 cm^−1^, vibrations of SiO_4_ occur. The bending of H_2_O occurs near 1600 cm^−1^. The stretching of H_2_O occurs near 3500 cm^−1^.

Figure 2a shows calculated and observed (rruff.info/R060064) IR spectra of Na_2_ZrSi_6_O_15_·3H_2_O. The spectra show good overall agreement. The part of the experimental spectrum from 400 to 850 cm^−1^ is due mostly to SiO_4_ bending vibrational modes perturbed by H_2_O librations. However, pure H_2_O librational modes also occur within the range. The group of peaks from 976 cm^−1^ to 1171 cm^−1^ is entirely due to SiO_4_ stretching vibrations. In [7], it was pointed out that stretching vibrations of the Si–O–Zr fragments were split into bands of Si–O and Zr–O: the doublet 1010 + 1032 and the triplet 627 + 648 + 681 cm^−1^, respectively. In the present calculation, we do not find this splitting. The stretching vibrations at about 1000–1050 cm^−1^ are attributed to the Si–O–Zr fragment as a whole. In the spectral region at 600–700 cm^−1^, all bands correspond to SiO_4_ bending vibrations, water librations or perturbed water SiO_4_ bending vibrations. The peak near 1590 cm^−1^ is due to H_2_O bending vibrations. The peak is split due to different sites of H_2_O in elpidite, and its position is shifted roughly by 40 cm^−1^ with respect to the experiment. The group of peaks near 3500 cm^−1^ is due to stretching vibrations of H_2_O. The calculated position of the absorption band is shifted by 40–50 cm^−1^ to the left if we consider high energy experimental peaks at 3503 and 3550 cm^−1^.

The calculated spectrum of Na_1.5_Ca_0.25_ZrSi_6_O_15_·2.75H_2_O is shown in Figure 2b. It well agrees with that of the Ca-rich elpidite studied in [7]. As in pure Na-elpidite, the range 400–800 cm^−1^ corresponds to SiO_4_ bending vibrations perturbed by H_2_O librations. The SiO_4_ stretching modes are located near 1000 cm^−1^, and in contrast to Na-elpidite, there are several intense peaks within the range 950–970 cm^−1^. The peaks corresponding to water bending vibrations are split with respect to those in Na-elpidite and are located between 1580 and 1605 cm^−1^. The stretching bands of H_2_O also become split and a variety of intense peaks occur between 3262 and 3605 cm^−1^.

The calculated IR spectrum of the hypothetical CaZrSi_6_O_15_·2H_2_O elpidite is shown in Figure 2c. In the part of H_2_O librations and SiO_4_ bending (from 400 to 768 cm^−1^) the calculated spectrum is very similar to that of pure Na-elpidite. However, the SiO4 stretching band is shifted to the lower frequencies with respect to both Na_2_ZrSi_6_O_15_·3H_2_O and Na_1.5_Ca_0.25_ZrSi_6_O_15_·2.75H_2_O, and occurs between 950 and 1166 cm^−1^. The H_2_O vibration peaks are unsplit in CaZrSi_6_O_15_·2H_2_O due to equivalent chemical environments for all H2O molecules. The calculated water bending peak locates at 1592 cm^−1^, symmetric stretching at 3424 cm^−1^ and asymmetric at 3471 cm^−1^.

## 5. Conclusions

The results of the research presented in this article highlight the significant potential of the ab initio calculations in studying natural compounds. Vibrational spectroscopy has proven to be an effective tool for identifying some anionic groups and neutral molecules (H_2_O) in microporous minerals. The use of a combination of SCXRD, EMPA, IR-spectroscopy, and ab initio calculations makes it possible to analyze the spectroscopic features of materials, taking into account their structural characteristics. This goal was achieved in this article using the example of natural microporous silicate elpidite.

A detailed crystal–chemical study of the mineral samples was carried out. The simplified formulas are Na_2_Zr[Si_6_O_15_]·3H_2_O and (Na_1+*y*_Ca*_x_*□_1−*x-y*_)*_Σ_*_=2_Zr[Si_6_O_15_]·(3−*x*)H_2_O (□–vacancy) for studied elpidite from Burpala and Khan-Bogdo, respectively. Na → Ca isomorphism in the structure of the latter involves two sodium positions and a water site. Comparison of geometrical and distortion parameters shows insignificant differences between them. Important information about the crystal structures was also obtained from analyses of local and general stability of the structures.

Through the use of ab initio calculations of IR spectra, accurate information on the absorption lines in the IR spectra of elpidite was obtained. It was shown that in the range of 400–800 cm^−1^, the IR absorption is due to the bending vibrations of SiO_4_ tetrahedra and SiO_4_–H_2_O. In addition, it was shown that the librational modes of water molecules are also present in this region. For the range 950–1200 cm^−1^, it was shown that it is due mainly to the framework (Zr-octahedra + Si-tetrahedra) stretching vibrations. The energies of bending and stretching vibrations of water molecules were reproduced precisely. The calculated spectra for Na_2_ZrSi_6_O_15_·3H_2_O, as well as for Na_1.5_Ca_0.25_ZrSi_6_O_15_·2.75H_2_O are in good agreement with the experimental ones. Furthermore, the crystal stability of the CaZrSi_6_O_15_·2H_2_O model of elpidite was predicted. We expect that water molecules will have a much weaker effect on the framework vibrations in this hypothetical compound. Therefore, ab initio calculation would be a useful method for the interpretation of infrared absorption spectra and the prediction of a new crystal–chemical stable form of natural microporous and zeolite-like minerals.

## Figures and Tables

**Table 1:** Selected data on single crystals, data collection and structure refinement parameters of the studied elpidite samples (ElB—elpidite from Burpala (Russia); ElKhB—elpidite from Khan-Bogdo (Mongolia)).

Crystal Data	ElB-1	ElB-2	ElKhB-1	ElKhB-2
*a* (Å) *b* (Å) *c* (Å)	7.1183(2) 14.6968(5) 14.6032(5)	7.1299(5) 14.7131(10) 14.6276(9)	7.1303(1) 14.6532(2) 14.6303(2)	7.1296(1) 14.6437(2) 14.6270(2)
*V* (Å^3^)	1527.73(5)	1534.48(9)	1528.60(2)	1527.11(2)
*Z*	4	4	4	4
Crystal dimensions (mm)	0.15 × 0.10 × 0.09	0.18 × 0.11 × 0.09	0.13 × 0.10 × 0.10	0.16 × 0.09 × 0.08
Structural formula	Na_2_Zr_1.03_[Si_6_O_15_]·3.02H_2_O	Na_1.98_Zr_1.04_[Si_6_O_15_]·2.91H_2_O	Na_1.38_Ca_0.33_Zr_1.02_[Si_6_O_15_]·2.64H_2_O	Na_1.09_Ca_0.47_Zr_1.02_[Si_6_O_15_]·2.52H_2_O
**Data Collection**				
Independent reflections	3014	4431	4058	4869
R_merging_ (R_(int)_) (%)	6.90	4.70	4.90	7.00
h_min_, h_max_	−10, 10	−12, 12	−10, 11	−12, 12
k_min_, k_max_	−22, 22	−25, 17	−24, 24	−26, 26
l_min_, l_max_	−21, 22	−24, 25	−24, 24	−25, 26
**Refinement**				
Space group	*Pbcm*	*Pbcm*	*Pbcm*	*Pbcm*
Reflections used in the refinement (I > 3σ_(I)_)	1672	2151	2147	2533
N. of refined parameters	148	148	149	149
*R*^a^ [on *F*] (%)	2.47	3.00	2.12	2.78
*R*_w_^b^ [on *F*] (%)	2.41	3.12	2.92	3.42
Goof ^c^	1.1174	1.1013	1.0560	1.0177
Δρ_min_/Δρ_max_ (e^−^/Å^3^)	−0.50/0.50	−0.87/0.66	−0.52/0.46	−1.12/0.86

^a^*R* = Σ[|F_o_| − |F_c_|]/Σ|F_o_|. ^b^ *R*_w_ = [Σ[*w*(*F_o_*^2^ − *F_c_*^2^)^2^]/Σ[*w*(*F*_o_^2^)^2^]]^1/2^; *w* = Chebyshev optimized weights. ^c^ Goodness-of-fit = [Σ[*w*(*F_o_*^2^ − *F_c_*^2^)^2^]/(*N* − *p*]^1/2^, where *N* and *p* are the number of reflections and parameters, respectively.

**Table 2:** Average chemical composition (wt.%) of the studied elpidite crystals.

Constituent	This Study				ElMSHil^(a)^	ElMSHil^(b)^	ElLov	ElKhB^(c)^
	ElB-1	ElB-2	ElKhB-1	ElKhB-2				
SiO_2_	60.2(6)	60.7(2)	60.4(7)	61.1(7)	59.82	58.45(53)	59.02	61.05
Al_2_O_3_	0.02(2)	0.01(1)	0.03(3)	0.04(2)	0.08	0.04(6)	0.15	—
Na_2_O	9.6(5)	9.5(6)	6.3(7)	5.5(5)	7.82	9.9(1)	10.17	8.64
MgO	0.02(1)	0.01(1)	b.d.l.	0.02(2)	0.01	0.01(1)	—	—
K_2_O	0.06(4)	0.12(3)	0.08(3)	0.06(4)	0.07	0.02(3)	0.04	0.08
CaO	0.03(1)	0.02(2)	2.9(3)	3.8(2)	0.62	0.01(1)	0.04	1.41
TiO_2_	0.01(1)	0.02(1)	0.07(5)	0.07(2)	1.15	0.1(1)	0.04	—
V_2_O_3_	b.d.l.	b.d.l.	b.d.l.	b.d.l.	—	0.03(5)	—	—
Cr_2_O_3_	b.d.l.	b.d.l.	b.d.l.	b.d.l.	—	0.06(8)	—	—
MnO	b.d.l.	0.02(2)	0.08(5)	b.d.l.	0.71	0.06(6)	—	—
FeO	0.04(4)	0.05(5)	0.36(5)	0.14(4)	0.89	—	—	—
Fe_2_O_3_	n.d.	n.d.	n.d.	n.d.	—	0.08(9)	0.02	—
NiO	b.d.l.	b.d.l.	b.d.l.	b.d.l.	—	0.03(6)	—	—
CuO	0.05(3)	0.08(7)	0.12(6)	0.06(4)	—	—	—	—
SrO	0.06(6)	0.02(2)	b.d.l.	b.d.l.	—	0.03(4)	—	—
ZrO_2_	19.3(6)	19.2(4)	19.4(5)	19.8(6)	15.00	20.0(4)	20.89	20.94
Nb_2_O_5_	b.d.l.	b.d.l.	b.d.l.	b.d.l.	1.12	0.2(2)	0.62	—
BaO	b.d.l.	b.d.l.	b.d.l.	b.d.l.	—	0.2(2)	—	—
La_2_O_3_	b.d.l.	0.04(4)	b.d.l.	b.d.l.	0.12	—	—	—
Ce_2_O_3_	0.09(9)	0.06(6)	0.08(7)	b.d.l.	0.24	—	—	—
Pr_2_O_3_	0.04(4)	0.10(6)	b.d.l.	b.d.l.	—	—	—	—
Nd_2_O_3_	0.06(6)	0.03(3)	0.06(6)	b.d.l.	—	—	—	—
Sm_2_O_3_	0.04(4)	b.d.l.	b.d.l.	0.09(9)	—	—	—	—
Eu_2_O_3_	b.d.l.	b.d.l.	b.d.l.	b.d.l.	—	—	—	—
Gd_2_O_3_	b.d.l.	b.d.l.	b.d.l.	b.d.l.	—	—	—	—
Dy_2_O_3_	0.1(1)	0.05(5)	b.d.l.	b.d.l.	—	—	—	—
Ho_2_O_3_	b.d.l.	b.d.l.	0.06(6)	0.12(9)	—	—	—	—
Er_2_O_3_	b.d.l.	b.d.l.	0.07(7)	0.07(7)	—	—	—	—
Yb_2_O_3_	0.08(8)	0.09(9)	0.2(1)	0.08(8)	—	—	—	—
Lu_2_O_3_	0.08(8)	b.d.l.	0.11(7)	b.d.l.	—	—	—	—
HfO_2_	0.8(1)	0.6(2)	0.5(1)	0.4(1)	0.11	—	0.43	—
F	b.d.l.	b.d.l.	b.d.l.	b.d.l.	0.11	0.04(7)	—	—
Cl	b.d.l.	b.d.l.	b.d.l.	b.d.l.	0.01	0.01(2)	—	—
P_2_O_5_	b.d.l.	b.d.l.	b.d.l.	b.d.l.	—	0.01(3)	—	—
								
Total	90.68	90.72	91.12	91.49	88.05	89.38	91.42	92.12

b.d.l. = below detection limit. n.d. = not detected. ElB—elpidite from Burpala (Russia) (this study); ElKhB—elpidite from Khan Bogdo (Mongolia) (this study); ElMSHil^(a)^—elpidite from Mt St. Hilaire (Canada) [34]; ElMSHil^(b)^—elpidite from Mt St. Hilaire (Canada) [15]; ElLov—elpidite from Mount Alluav, Lovozero (Russia) [14]; ElKhB^(c)^—elpidite from Khan Bogdo (Mongolia) [7].

**Table 3:** The most intense peaks (>10% of maximal intensity) in simulated IR absorption spectra of elpidite models Na_2_ZrSi_6_O_15_·3H_2_O, Na_1.5_Ca_0.25_ZrSi_6_O_15_·2.75H_2_O and hypothetical elpidite model CaZrSi_6_O_15_·2H_2_O. All peaks related to H_2_O vibrations are shown.

Wavenumber (cm^−1^)	Absorbance (arb. Units)	Peak Attribution	Wavenumber (cm^−1^)	Absorbance (arb. Units)	Peak Attribution	Wavenumber (cm^−1^)	Absorbance (arb. Units)	Peak Attribution
Na_2_ZrSi_6_O_15_·3H_2_O Model			Na_1.5_Ca_0.25_ZrSi_6_O_15_·2.75H_2_O Model			CaZrSi_6_O_15_·2H_2_O Model		
401–420	0.12–0.39	Si1 + Si2 + Si3 + W1	406–409	0.33–0.72	Si1 + Si2 + Si3 + W1 + W2	411	0.35	framework + W1
480	0.11	framework + W1 + W2	419	0.16	framework +W1	561	0.15	W1
574	0.14	W1 + W2	482	0.17	framework + W1 + W2	615	0.1	framework + W1
609	0.12	framework + W1 + W2	501–620	0.10–0.45	W1 + W2	632–768	0.09–0.11	framework
623	0.26	W1 + W2	761	0.34	Si1 + Si2 + Si3 + W1 + W2	950–1005	0.12–1.00	framework
762	0.12	Si1 + Si2 + Si3 + W1 + W2	790	0.14	Si1 + Si3 + W1 + W2	1032	0.33	Si1 + Si2
976	0.3	Si2 + Si3	948	0.22	Si1 + Si3	1061	0.14	framework + W1
982	0.73	framework	953	0.42	Si1 + Si2 + Si3	1085	0.12–1.00	Si1 + Si3
986–999	0.13–0.5	Zr + Si1 + Si3	960	0.73	Si1 + Si3	1139	0.25	Si1 + Si2 + Si3
1010	1	framework	966	0.19	Si2 + Si3	1166	0.21	Si1 + Si3
1015	0.47	Zr + Si1 + Si3	973–978	0.33–0.66	Si1 + Si2 + Si3	1592	0.08	W1
1021–1022	0.33–0.70	Si1 + Si2 + Si3	981	0.65	Si1 + Si3	3420–3424	0.06–0.25	W1
1030	0.75	framework	985–998	0.17–0.84	framework	3471	0.53	W1
1074–1084	0.11–0.17	framework + W1	1000	0.16	Zr + Si1 + Si3			
1122	0.21	Si2	1000–1029	0.11–1.00	framework			
1171–1182	0.10–0.29	framework	1031	0.36	Si1 + Si2			
1589	0.13	W1 + W2	1054–1094	0.08–0.21	framework + W1			
1592	0.01	W2	1125	0.67	Si2			
1594	0.02	W1 + W2	1165	0.11	framework			
3312–3347	0.08–0.50	W2	1167–1170	0.10–0.16	Si1 + Si3			
3459–3465	0.16–0.24	W1 + W2	1173	0.13	Si1 + Si2 + Si3			
3517–3519	0.03–0.50	W1	1178	0.45	framework			
			1584–1589	0.01–0.19	W1 + W2			
			1591	0.02	W2			
			1597	0.05	W1 + W2			
			1599	0.06	W1			
			1604	0.01	W1 + W2			
			3262–3308	0.31–0.34	W2			
			3315	0.34	W1 + W2			
			3327–3367	0.23–0.40	W2			
			3385–3467	0.16–0.40	W1 + W2			
			3473	0.28	W1			
			3482–3487	0.06–0.24	W1 + W2			
			3491–3517	0.18–0.32	W1			
			3519	0.27	W2			
			3532–3605	0.20–0.28	W1			

**Table 4:** The global instability index (GII, %) calculated for the crystal structure of elpidite under study (ElB—elpidite from Burpala (Russia); ElKhB—elpidite from Khan-Bogdo (Mongolia)).

Sample	GII (%) Zr	GII (%) Na	GII (%) Si	GII (%) O	GII (%) Total
ElB-1	2.60	4.77	11.02	9.82	9.26
ElB-2	1.40	5.81	9.02	9.47	8.65
ElKhB-1	1.20	6.20	13.13	10.80	10.48
ElKhB-2	0.40	12.79	12.92	10.80	11.19

## Data Availability

Data sharing is not applicable for this article.

## Supplementary Materials

The following are available online at https://www.mdpi.com/article/10.3390/ma14092160/s1, Figure S1: Bond length distortion (BLD) for tetrahedral of elpidite crystal structure against tetrahedral angle variance (TAV). 1 and 2 are ElB1 and ElB2 – elpidite from Burpala (Russia); 3 and 4 are ElKhB1and ElKhB2 – elpidite from Khan-Bogdo (Mongolia)., Table S1: comparative crystallographic data for elpidite, previously published, Table S2: crystallographic coordinates, occupancies and equivalent/isotropic atomic displacement parameters (Å^2^) of elpidite sample ElB-1, Table S3: anisotropic atomic displacement parameters (Å^2^) of elpidite sample ElB-1, Table S4: crystallographic coordinates, occupancies and equivalent/isotropic atomic displacement parameters (Å^2^) of elpidite sample ElB-2, Table S5: anisotropic atomic displacement parameters (Å^2^) of elpidite sample ElB-2, Table S6: crystallographic coordinates, occupancies and equivalent/isotropic atomic displacement parameters (Å^2^) of elpidite sample ElKhB-1, Table S7: anisotropic atomic displacement parameters (Å^2^) of elpidite sample ElKhB-2, Table S8: crystallographic coordinates, occupancies and equivalent/isotropic atomic displacement parameters (Å^2^) of elpidite sample ElKhB-2, Table S9: anisotropic atomic displacement parameters (Å^2^) of elpidite sample ElKhB-2, Table S10: selected bond distances (Å) for tetrahedra and polyhedra of the studied elpidite samples, Table S11: selected angles (º) for tetrahedra and polyhedra of the studied elpidite samples, Table S12: calculated geometrical parameters for polyhedra in the crystal structures of studied elpidite samples, Table S13: calculated distortion parameters for polyhedra in the crystal structures of studied elpidite samples, Table S14: valence balance calculation for studied elpidite sample (ElB-1), Table S15: valence balance calculation for studied elpidite sample (ElB-2), Table S16: valence balance calculation for studied elpidite sample (ElKhB-1), Table S17: valence balance calculation for studied elpidite sample (ElKhB-2), Table S18: crystallographic coordinates of the modeled elpidites, Table S19: the positions of the bands (cm^−1^) in the IR spectra of elpidite from literature, Table S20: calculated vibrational modes in simulated structure models of elpidite, Table S21: comparative geometrical parameters for tetrahedra and polyhedra in the crystal structures of studied samples and simulated models of Na_2_ZrSi_6_O_15_·3H_2_O and Na_1.5_Ca_0.25_ZrSi_6_O_15_·2.75H_2_O elpidite, Table S22: calculated geometrical parameters for tetrahedra and polyhedra in the crystal structure of the simulated model of hypothetical CaZrSi_6_O_15_·2H_2_O elpidite, Figure S1: bond length distortion (BLD) for tetrahedral of elpidite crystal structure against tetrahedral angle variance (TAV), Figure S2: edge length distortion (ELD) for tetrahedral of elpidite crystal structure against tetrahedral angle variance (TAV), Figure S3: the volume of the coordination polyhedra (Vp) of elpidite crystal structure against polyhedra volume sphericity (SPHv).
