# Supplementary material for: New Insights into the Crystal Chemistry of Elpidite, Na2Zr[Si6O15]·3H2O and (Na1+YCax□1−X−Y)Σ=2Zr[Si6O15]·(3−X)H2O, and Ab Initio Modeling of IR Spectra
Source: Materials (Basel). 2021 Apr 23;14(9):2160. doi: 10.3390/ma14092160 (PMC8122974; doi:10.3390/ma14092160)
Supplement: Supplementary file 1 [file materials-14-02160-s001.zip › materials-1179982-sup1.pdf]

materials

New insights into the crystal chemistry of elpidite, $Na_2Zr[Si_6O_{15}]\cdot 3H_2O$ and $(Na_{1+y}Ca_x\square_{1-x-y})_{\Sigma=2}Zr[Si_6O_{15}]\cdot(3-x)H_2O$, and *ab initio* modeling of IR spectra

Alexander Bogdanov[1], Ekaterina Kaneva[1,2]* and Roman Shendrik[1]

[1] Vinogradov Institute of Geochemistry, Siberian Branch of the Russian Academy of Sciences, 664033 Irkutsk, Russia; alex.bogdanov2012@gmail.com (A.B.) r.shendrik@gmail.com (R.S.)
[2] Department of Subsoil Use, Irkutsk National Research Technical University, 664074 Irkutsk, Russia
* Correspondence: kev604@mail.ru

Table S1. Comparative crystallographic data for elpidite, previously published.

Authors	Occurrence	Chemical Formula	Unit Cell Parameters			Space Group
			a (Å)	*b* (Å)	*c* (Å)	
[1,2]	Lovozero, Kola Peninsula, Russia	$Na_2ZrSi_6O_{15}\cdot 3H_2O$	7.4	14.4	7.05	*Pbm*2
[3]	Mont Saint Hilaire, Quebec, Canada	$Na_{1.04}K_{0.01}Zr_{1.05}Si_6O_{15}\cdot 3H_2O$	7.14	14.68	14.58	*Pbmm*
[4]	Mont Saint Hilaire, Quebec, Canada	$Na_2ZrSi_6O_{15}\cdot 3H_2O$	7.14(2)	14.68(1)	14.65(1)	*Pbcm*
[5]	Khan-Bogdo, Mongolia	$(Na_{1.31}Ca_{0.38}K_{0.03}Mg_{0.03})_{1.75}ZrSi_6O_{15}\cdot 2.9H_2O$	7.31	14.68	7.13	*Pbm*2
[6]	Lovozero, Kola Peninsula, Russia	$(Na_{1.98}K_{0.01})(Zr_{1.02}Nb_{0.03}Hf_{0.01})(Si_{5.92}Al_{0.02})O_{15}\cdot 3.28H_2O$	7.1136(1)	14.6764(2)	14.5977(2)	*Pbcm*
[7]	Khan-Bogdo, Mongolia	$Na_{1.65}Ca_{0.15}K_{0.01}(H_{0.03})ZrSi_6O_{15}\cdot nH_2O$, where n ≈ 3.	7.131(1)	14.685(1)	14.635(2)	*Pbcm*
[8]	Khan-Bogdo, Mongolia	–	7.1320(2)	14.6787(4)	14.6297(3)	*Pbcm*
[9]	Narssarssuk, Greenland	$Na_2ZrSi_6O_{15}\cdot 3H_2O$	7.14	14.68	14.65	*Pbcm*
[10]	Mont Saint Hilaire, Quebec, Canada	$Na_{1.96}ZrSi_{5.97}O_{15}\cdot 2.9H_2O$	7.1134(1)	14.6796(2)	14.6030(2)	*Pbcm*
[11]	Lovozero, Kola Peninsula, Russia	$(Na_{1.98}K_{0.01})(Zr_{1.02}Nb_{0.03}Hf_{0.01})(Si_{5.92}Al_{0.02})O_{15}\cdot 3.40H_2O$	14.6127(7)	7.3383(4)	7.1148(3)	*Pma*2
[12]	Lovozero, Kola Peninsula, Russia	–	7.0956(5)	14.6536(6)	14.5696(7)	*Pbcm*
[13]	Khibiny, Kola Peninsula, Russia	$(Na_{1.05}K_{0.08}Ca_{0.01})(H_3O)_{0.74}(Zr_{0.89}Ti_{0.10}Nb_{0.03})Si_6O_{15}\cdot 3.47H_2O$	14.5916(6)	7.3294(3)	7.1387(2)	*Pma*2

Table S2. Crystallographic coordinates, occupancies and equivalent/isotropic atomic displacement parameters (Å^2) of elpidite sample ElB-1.

Site	x/a	y/b	z/c	Occ.	Ueq
Zr	0.49564(6)	0.25	0.5	1.035(3)	0.0084
Si1	0.7721(1)	0.38579(5)	0.64609(6)	1.00(6)	0.0096
Si2	0.5077(1)	0.04737(4)	0.64171(4)	1.00(6)	0.0093
Si3	0.2187(1)	0.39216(5)	0.64406(6)	1.00(6)	0.0087
O1	0.9945(3)	0.4042(1)	0.6392(1)	1.00(7)	0.0156
O2	0.7179(4)	0.3550(2)	0.75	1.0(1)	0.0148
O3	0.7079(3)	0.3090(2)	0.5771(2)	1.00(7)	0.0139
O4	0.6764(3)	0.4840(2)	0.6255(2)	1.00(8)	0.0164
O5	0.5251(4)	0.0712(2)	0.75	1.0(1)	0.0156
O6	0.4905(4)	0.1406(1)	0.5883(1)	1.00(7)	0.0152
O7	0.3055(3)	0.4899(1)	0.6117(2)	1.00(7)	0.0134
O8	0.2838(4)	0.3766(2)	0.75	1.0(1)	0.0129
O9	0.2904(3)	0.3097(2)	0.5828(2)	1.00(7)	0.0151
Na1	0.4424(3)	0.2315(1)	0.75	1.014(9)	0.0332
Na2	−0.0045(3)	0.25	0.5	0.990(7)	0.0213
Ow1	0.0094(4)	0.1130(2)	0.5811(2)	0.995(8)	0.0308
Ow2	0.1225(7)	0.1889(3)	0.75	1.03(1)	0.0474

Table S3. Anisotropic atomic displacement parameters (Å^2) of elpidite sample ElB-1.

Site	U11	U22	U33	U23	U13	U12
Zr	0.0082(1)	0.0079(1)	0.0090(1)	−0.0002(1)	0.0000	0.0000
Si1	0.0065(3)	0.0107(3)	0.0116(5)	−0.0010(3)	−0.0004(3)	0.0003(2)
Si2	0.0090(2)	0.0084(2)	0.0106(2)	0.0012(2)	−0.0001(4)	0.0003(3)
Si3	0.0064(3)	0.0096(3)	0.0102(4)	−0.0013(3)	0.0001(3)	0.0000(2)
O1	0.0076(6)	0.0194(8)	0.0198(8)	−0.0004(6)	0.001(1)	−0.0012(9)
O2	0.014(1)	0.019(2)	0.011(1)	0.0000	0.0000	−0.004(1)
O3	0.0131(9)	0.0155(9)	0.013(1)	−0.0053(9)	−0.0043(9)	−0.0001(8)
O4	0.0137(9)	0.0122(9)	0.023(1)	0.0025(9)	0.0027(2)	0.0028(7)
O5	0.021(2)	0.014(1)	0.012(1)	0.0000	0.0000	0.001(1)
O6	0.0155(8)	0.0112(7)	0.0190(7)	0.0048(6)	0.002(1)	0.0007(9)
O7	0.0136(9)	0.0119(2)	0.0147(9)	0.0019 (7)	−0.0033(7)	−0.0031(7)
O8	0.013(1)	0.015(1)	0.011(1)	0.0000	0.0000	0.001(1)
O9	0.0136(9)	0.0135(9)	0.018(1)	−0.0019(9)	0.002(1)	0.0023(8)
Na1	0.037(1)	0.0156(9)	0.047(1)	0.0000	0.0000	0.0059(7)
Na2	0.0162(7)	0.0210(7)	0.0269(8)	−0.0014(7)	0.0000	0.0000
Ow1	0.027(1)	0.025(1)	0.041(1)	0.009(1)	−0.003(1)	−0.005(1)
Ow2	0.055(3)	0.048(3)	0.040(2)	0.0000	0.0000	−0.018(2)

Table 4. Crystallographic coordinates, occupancies and equivalent/isotropic atomic displacement parameters (Å^2) of elpidite sample ElB-2.

Site	x/a	y/b	z/c	Occ.	Ueq
Zr	0.49571(5)	0.25	0.5	1.036(3)	0.0087
Si1	0.7722(1)	0.38596(5)	0.64607(6)	1.00(6)	0.0093
Si2	0.5073(1)	0.04735(4)	0.64167(4)	1.00(7)	0.0093
Si3	0.2186(1)	0.39210(5)	0.64403(6)	1.00(6)	0.0094

O1	0.9947(3)	0.4041(1)	0.6391(1)	1.00(8)	0.0162
O2	0.7187(4)	0.3553(2)	0.75	1.0(1)	0.0156
O3	0.7084(3)	0.3092(2)	0.5770(2)	1.00(8)	0.0161
O4	0.6758(3)	0.4842(1)	0.6251(2)	1.00(9)	0.0175
O5	0.5247(4)	0.0710(2)	0.75	1.0(1)	0.0169
O6	0.4910(3)	0.1403(1)	0.5884(1)	1.00(8)	0.0154
O7	0.3058(3)	0.4898(1)	0.6119(2)	1.00(8)	0.0142
O8	0.2836(4)	0.3762(2)	0.75	1.0(1)	0.0135
O9	0.2907(3)	0.3099(1)	0.5824(2)	1.00(8)	0.0147
Na1	0.4439(3)	0.2316(1)	0.75	0.99(1)	0.0329
Na2	−0.0045(3)	0.25	0.5	0.994(8)	0.0214
Ow1	0.0094(4)	0.1131(2)	0.5810(2)	0.973(9)	0.0299
Ow2	0.1226(7)	0.1889(4)	0.75	0.96(2)	0.0450

Table 5. Anisotropic atomic displacement parameters (Å^2) of elpidite sample ElB-2.

Site	U11	U22	U33	U23	U13	U12
Zr	0.0090(1)	0.00780(9)	0.00930(9)	−0.00006(9)	0.0000	0.0000
Si1	0.0069(3)	0.0106(3)	0.0103(4)	−0.0015(3)	−0.0006(3)	0.0006(2)
Si2	0.0091(2)	0.0080(2)	0.0109(2)	0.0012(2)	−0.0003(3)	0.0004(2)
Si3	0.0073(3)	0.0096(3)	0.0112(4)	−0.0009(3)	0.0001(3)	0.0001(2)
O1	0.0069(6)	0.0207(7)	0.0210(7)	−0.0013(6)	0.002(1)	−0.0002(8)
O2	0.016(1)	0.020(1)	0.011(1)	0.0000	0.0000	−0.002(1)
O3	0.016(1)	0.0142(8)	0.018(1)	−0.0045(8)	−0.0031(9)	−0.0022(8)
O4	0.0142(9)	0.0143(8)	0.024(1)	0.0012(8)	0.0007(8)	0.0062(7)
O5	0.022(2)	0.017(1)	0.0110(9)	0.0000	0.0000	0.004(1)
O6	0.0185(8)	0.0100(6)	0.0178(7)	0.0040(5)	0.000(1)	0.0018(8)
O7	0.0137(8)	0.0113(8)	0.0176(9)	0.0017(7)	−0.0044(7)	−0.0041(6)
O8	0.014(1)	0.015(1)	0.012(1)	0.0000	0.0000	−0.0002(9)
O9	0.0146(9)	0.0137(8)	0.016(1)	−0.0033(8)	0.0025(9)	0.0021(7)
Na1	0.043(1)	0.0148(9)	0.041(1)	0.0000	0.0000	0.0060(8)
Na2	0.0177(7)	0.0201(7)	0.0264(8)	−0.0024(7)	0.0000	0.0000
Ow1	0.025(1)	0.024(1)	0.040(1)	0.011(1)	−0.003(1)	0.002(1)
Ow2	0.055(3)	0.046(3)	0.034(2)	0.0000	0.0000	−0.021(2)

Table 6. Crystallographic coordinates, occupancies and equivalent/isotropic atomic displacement parameters (Å^2) of elpidite sample ElKhB-1.

Site	x/a	y/b	z/c	Occ.	Ueq
Zr	0.49668(4)	0.25	0.5	1.0232(9)	0.0076
Si1	0.77341(7)	0.38608(4)	0.64617(4)	1.0000(7)	0.0092
Si2	0.50611(8)	0.04743(3)	0.64196(3)	1.0000(7)	0.0088
Si3	0.21942(7)	0.39118(4)	0.64446(4)	1.0000(7)	0.0090
O1	0.9960(2)	0.4036(1)	0.6385(1)	1.0000(7)	0.0171
O2	0.7203(3)	0.3559(2)	0.75	1.0000(7)	0.0172
O3	0.7100(2)	0.3089(1)	0.5772(1)	1.0000(7)	0.0168
O4	0.6779(2)	0.4843(1)	0.6247(1)	1.0000(7)	0.0174
O5	0.5193(3)	0.0715(1)	0.75	1.0000(7)	0.0171
O6	0.4926(2)	0.13969(9)	0.5874(1)	1.0000(7)	0.0171
O7	0.3073(2)	0.4891(1)	0.6136(1)	1.0000(7)	0.0151
O8	0.2816(3)	0.3737(2)	0.75	1.0000(7)	0.0158

O9	0.2888(2)	0.3098(1)	0.5815(1)	1.0000(7)	0.0170
Na1	0.4416(3)	0.2313(1)	0.75	0.775(1)	0.0312
Na2	−0.0028(2)	0.25	0.5	0.605(1)	0.0230
Ca2	−0.0028(2)	0.25	0.5	0.330(1)	0.0230
Ow1	0.0078(3)	0.1135(1)	0.5822(2)	0.947(1)	0.0323
Ow2	0.1206(6)	0.1883(3)	0.75	0.747(1)	0.0410

Table 7. Anisotropic atomic displacement parameters (Å[2]) of elpidite sample ElKhB-2.

Site	U11	U22	U33	U23	U13	U12
Zr	0.00880(8)	0.00665(8)	0.00735(8)	−0.00010(7)	0.0000	0.0000
Si1	0.0078(2)	0.0099(2)	0.0099(3)	−0.0018(2)	−0.0006(2)	0.0003(2)
Si2	0.0106(2)	0.0072(2)	0.0086(2)	0.0012(1)	0.0000(3)	0.0000(2)
Si3	0.0073(2)	0.0102(2)	0.0095(3)	−0.0019(2)	0.0005(2)	−0.0002(2)
O1	0.0069(5)	0.0231(7)	0.0214(7)	−0.0011(5)	−0.0008(7)	0.0000(6)
O2	0.018(1)	0.022(2)	0.012(1)	0.0000	0.0000	−0.0032(9)
O3	0.0192(7)	0.0156(7)	0.0156(9)	−0.0054(6)	−0.0057(7)	−0.0026(6)
O4	0.0160(7)	0.0130(7)	0.0231(9)	0.0007(6)	0.0024(6)	0.0049(5)
O5	0.027(1)	0.0156(8)	0.0085(8)	0.0000	0.0000	0.0007(9)
O6	0.0224(7)	0.0107(5)	0.0182(6)	0.0060(5)	−0.0007(8)	0.0009(7)
O7	0.0156(7)	0.0126(7)	0.0171(8)	0.0020(6)	−0.0023(6)	−0.0048(5)
O8	0.016(1)	0.021(1)	0.010(1)	0.0000	0.0000	0.0040(8)
O9	0.0190(7)	0.0135(7)	0.019(1)	−0.0027(6)	0.0058(7)	0.0027(6)
Na1	0.042(1)	0.0123(8)	0.040(1)	0.0000	0.0000	0.0065(8)
Na2	0.0172(4)	0.0224(4)	0.0292(5)	−0.0021(4)	0.0000	0.0000
Ca2	0.0172(4)	0.0224(4)	0.0292(5)	−0.0021(4)	0.0000	0.0000
Ow1	0.0271(9)	0.0268(9)	0.043(1)	0.0142(8)	−0.004(1)	−0.0020(9)
Ow2	0.053(3)	0.037(2)	0.033(2)	0.0000	0.0000	−0.012(2)

Table 8. Crystallographic coordinates, occupancies and equivalent/isotropic atomic displacement parameters (Å[2]) of elpidite sample ElKhB-2.

Site	x/a	y/b	z/c	Occ.	Ueq
Zr	0.49708(4)	0.25	0.5	1.024(2)	0.0081
Si1	0.77403(7)	0.38645(3)	0.64596(4)	1.000(7)	0.0098
Si2	0.50513(8)	0.04744(3)	0.64198(3)	1.000(7)	0.0094
Si3	0.21963(7)	0.39088(3)	0.64452(4)	1.000(7)	0.0098
O1	0.9961(2)	0.4035(1)	0.6381(1)	1.000(7)	0.0190
O2	0.7209(3)	0.3569(2)	0.75	1.000(7)	0.0185
O3	0.7105(2)	0.3089(1)	0.5772(1)	1.000(7)	0.0179
O4	0.6791(2)	0.4848(1)	0.6238(2)	1.000(7)	0.0187
O5	0.5169(4)	0.0713(1)	0.75	1.000(7)	0.0190
O6	0.4935(3)	0.13967(8)	0.58718(9)	1.000(7)	0.0182
O7	0.3080(2)	0.4889(1)	0.6146(1)	1.000(7)	0.0167
O8	0.2811(3)	0.3725(2)	0.75	1.000(7)	0.0185
O9	0.2882(2)	0.3094(1)	0.5810(1)	1.000(7)	0.0178
Na1	0.4410(4)	0.2311(1)	0.75	0.686(6)	0.0313
Na2	−0.0022(2)	0.25	0.5	0.399(9)	0.0259
Ca2	−0.0022(2)	0.25	0.5	0.473(6)	0.0259
Ow1	0.0064(3)	0.1142(2)	0.5822(2)	0.946(6)	0.0359

Ow2	0.1222(8)	0.1882(4)	0.75	0.634(8)	0.0431

Table 9. Anisotropic atomic displacement parameters (Å^2) of elpidite sample ElKhB-2.

Site	U11	U22	U33	U23	U13	U12
Zr	0.00908(7)	0.00778(7)	0.00755(7)	−0.00021(5)	0.0000	0.0000
Si1	0.0077(2)	0.0117(2)	0.0100(3)	−0.0024(2)	−0.0008(2)	0.0002(2)
Si2	0.0110(2)	0.0082(1)	0.0090(2)	0.0012(1)	0.0000(2)	0.0002(2)
Si3	0.0076(2)	0.0116(2)	0.0102(3)	−0.0023(2)	0.0004(2)	−0.0004(2)
O1	0.0073(4)	0.0259(6)	0.0238(6)	−0.0005(4)	−0.0005(7)	−0.0005(6)
O2	0.0172(9)	0.027(2)	0.012(1)	0.0000	0.0000	−0.0024(8)
O3	0.0210(6)	0.0167(6)	0.0161(9)	−0.0041(6)	−0.0051(6)	−0.0038(6)
O4	0.0165(6)	0.0145(6)	0.0250(9)	0.0007(6)	0.0015 (6)	0.0063(5)
O5	0.030(1)	0.0171(7)	0.0103(6)	0.0000	0.0000	0.0019(8)
O6	0.0255(6)	0.0111(4)	0.0180(5)	0.0063(4)	0.0000(8)	0.0011(6)
O7	0.0164(6)	0.0154(6)	0.0182(7)	0.0009(5)	−0.0023(5)	−0.0053(5)
O8	0.0176(9)	0.027(1)	0.011(1)	0.0000	0.0000	0.0040(8)
O9	0.0211(6)	0.0145(6)	0.0179(9)	−0.0042(6)	0.0082(7)	0.0022(5)
Na1	0.044(1)	0.0123(8)	0.038(1)	0.0000	0.0000	0.0077(8)
Na2	0.0183(3)	0.0249(4)	0.0343(5)	−0.0043(3)	0.0000	0.0000
Ca2	0.0183(3)	0.0249(4)	0.0343(5)	−0.0043(3)	0.0000	0.0000
Ow1	0.0288(9)	0.0308(9)	0.048(1)	0.0152(8)	−0.005(1)	−0.0025(9)
Ow2	0.051(3)	0.039(3)	0.040(3)	0.0000	0.0000	−0.015(2)

Table 10. Selected bond distances (Å) for tetrahedra and polyhedra of the studied elpidite samples.

	ElB-1	ElB-2	ElKhB-1	ElKhB-2
Si1-O1	1.609(3)	1.612(2)	1.611(1)	1.610(2)
Si1-O2	1.630(1)	1.631(1)	1.627(1)	1.627(1)
Si1-O3	1.580(3)	1.583(2)	1.581(2)	1.583(2)
Si1-O4	1.625(3)	1.629(2)	1.623(2)	1.624(2)
<Si1-O>	1.611(6)	1.614(4)	1.611(2)	1.611(4)
Si2-O4	1.623(3)	1.621(2)	1.625(2)	1.624(2)
Si2-O5	1.6245(9)	1.6271(9)	1.6221(6)	1.6203(6)
Si2-O6	1.581(2)	1.579(2)	1.573(1)	1.573(1)
Si2-O7	1.636(3)	1.638(2)	1.635(2)	1.635(2)
<Si2-O>	1.616(5)	1.616(4)	1.614(2)	1.613(2)
Si3-O1	1.607(3)	1.608(2)	1.606(1)	1.607(2)
Si3-O7	1.634(3)	1.635(2)	1.629(1)	1.627(2)
Si3-O8	1.631(1)	1.635(1)	1.6267(9)	1.6263(9)
Si3-O9	1.590(3)	1.594(2)	1.585(2)	1.589(2)
<Si3-O>	1.616(6)	1.618(4)	1.612(2)	1.612(4)
Zr-O3 $^{(\times2)}$	2.074(3)	2.080(2)	2.082(2)	2.082(2)
Zr-O6 $^{(\times2)}$	2.062(2)	2.068(2)	2.061(1)	2.058(1)
Zr-O9 $^{(\times2)}$	2.090(3)	2.090(2)	2.095(2)	2.093(2)
<Zr-O >	2.075(7)	2.079(5)	2.079(4)	2.078(4)

Na1-O2	2.672(4)	2.675(4)	2.699(3)	2.715(4)
Na1-O5	2.429(4)	2.431(3)	2.407(3)	2.402(3)
Na1-O6 (×2)	2.734(3)	2.739(2)	2.755(2)	2.758(2)
Na1-O8	2.413(4)	2.416(4)	2.378(5)	2.364(4)
Na1-O9 (×2)	2.907(3)	2.921(3)	2.930(2)	2.935(2)
Na1-Ow2	2.362(6)	2.375(6)	2.374(5)	2.358(7)
<Na1-O >	2.645(9)	2.652(8)	2.654(8)	2.653(9)
Na2-O3 (×2)	2.492(3)	2.493(3)	2.493(2)	2.493(2)
Na2-O9 (×2)	2.577(3)	2.581(3)	2.552(2)	2.540(2)
Na2-Ow1 (×2)	2.338(3)	2.339(2)	2.334(2)	2.325(2)
<Na2-O >	2.469(7)	2.471(5)	2.460(4)	2.453(4)

Table 11. Selected angles (º) for tetrahedra and polyhedra of the studied elpidite samples.

	ElB-1	ElB-2	ElKhB-1	ElKhB-2
O1-Si1-O2	109.74(8)	109.57(8)	109.72(6)	109.72(6)
O1-Si1-O3	111.4(1)	111.2(1)	110.56(8)	110.36(8)
O1-Si1-O4	104.6(1)	104.9(1)	104.97(8)	105.00(8)
O2-Si1-O3	109.1(1)	109.3(1)	109.56(7)	109.69(8)
O2-Si1-O4	108.7(1)	108.8(1)	108.91(7)	109.03(8)
O3-Si1-O4	113.3(1)	113.1(1)	113.00(9)	112.93(9)
<O-Si1-O>	109.5(2)	109.5(2)	109.5(2)	109.5(2)
O4-Si2-O5	109.1(1)	109.2(1)	108.78(7)	108.87(8)
O4-Si2-O6	111.2(1)	111.3(1)	111.15(8)	111.05(8)
O4-Si2-O7	108.7(1)	108.6(1)	108.66(8)	108.80(8)
O5-Si2-O6	107.40(8)	107.49(8)	108.15(6)	108.34(5)
O5-Si2-O7	108.08(9)	107.95(9)	108.30(7)	108.07(7)
O6-Si2-O7	112.2(1)	112.1(1)	111.74(8)	111.63(8)
<O-Si2-O>	109.4(2)	109.4(2)	109.5(2)	109.5(2)
O1-Si3-O7	105.4(1)	105.6(1)	105.47(8)	105.48(8)
O1-Si3-O8	108.86(8)	109.86(8)	109.85(6)	109.96(6)
O1-Si3-O9	112.2(1)	112.2(1)	111.31(8)	110.95(8)
O7-Si3-O8	106.85(9)	106.91(9)	107.29(7)	107.28(7)
O7-Si3-O9	112.7(1)	112.4(1)	112.39(9)	112.66(8)
O8-Si3-O9	109.6(1)	109.7(1)	110.35(7)	110.33(7)
<O-Si3-O>	109.3(2)	109.4(2)	109.4(2)	109.4(2)
O3-Zr-O3	86.50(9)	86.37(9)	86.14(9)	86.07(6)
O3-Zr-O6 (×2)	91.52(9)	91.37(8)	91.26(6)	91.12(6)
O3-Zr-O6 (×2)	89.96(9)	89.99(8)	89.93(7)	89.92(6)
O3-Zr-O9 (×2)	91.11(9)	91.22(9)	91.98(6)	92.34(6)
O6-Zr-O9 (×2)	87.30(9)	87.54(8)	87.98(7)	88.10(6)
O6-Zr-O9 (×2)	91.28(9)	91.16(8)	90.87(6)	90.89(6)
O9-Zr-O9	91.30(9)	91.22(9)	89.92(9)	89.27(6)
<O-Zr-O>	90.0(3)	90.0(3)	90.0(3)	90.0(2)
O2-Na1-O6 (×2)	103.89(4)	104.11(4)	103.44(3)	103.27(3)

O2-Na1-O9 (×2)	90.27(4)	90.31(4)	90.46(3)	90.45(3)
O5-Na1-O6 (×2)	59.70(4)	59.65(4)	59.71(3)	59.76(3)
O6-Na1-O9 (×2)	60.97(6)	60.99(6)	60.94(5)	60.84(4)
O6-Na1-Ow2 (×2)	89.49(6)	89.35(4)	89.88(3)	90.08(4)
O8-Na1-O9 (×2)	58.43(5)	58.36(4)	58.47(3)	58.57(3)
O9-Na1-Ow2 (×2)	75.28(4)	75.15(4)	75.27(3)	75.31(3)
O2-Na1-O5	118.8(1)	119.2(1)	119.3(1)	119.7(1)
O2-Na1-O8	75.1(1)	75.3(1)	76.1(1)	76.1(1)
O5-Na1-Ow2	88.6(1)	88.4(2)	87.9(1)	87.6(2)
O8-Na1-Ow2	77.5(1)	77.1(2)	76.7(1)	76.6(2)
<O-Na1-O >	79.8(4)	79.8(4)	79.8(4)	79.8(4)
O3-Na2-O9 (×2)	109.79(7)	109.84(8)	109.78(5)	109.88(5)
O3-Na2-Ow1 (×2)	96.07(8)	96.13(8)	95.14(6)	94.80(7)
O3-Na2-Ow1 (×2)	87.93(8)	87.87(8)	87.90(6)	87.70(7)
O9-Na2-Ow1 (×2)	84.83(8)	84.71(8)	85.43(6)	85.79(7)
O9-Na2-Ow1 (×2)	91.21(8)	91.32(8)	91.56(6)	91.74(7)
O3-Na2-O3	69.54(7)	69.62(7)	69.54(5)	69.49(5)
O9-Na2-O9	70.90(7)	70.71(7)	70.91(5)	70.76(5)
<O-Na2-O >	90.0(2)	90.0(2)	90.0(2)	90.0(2)

Table 12. Calculated geometrical parameters for polyhedra in the crystal structures of studied elpidite samples. BVS-bond-valence sum [14], ECoN-effective coordination number [15–18], Vp–a volume of the coordination polyhedron [19,20], r_v-average distance from the volume center to the ligands [19,20], Δ_v – a distance of the central atom to the volume center [19,20], r_s-average distance from the centroid to the ligands [19,20], Vs–a volume of the sphere fitted to the positions of ligands [19,20], ECCv-volume eccentricity [19,20], SPHv–volume sphericity [19,20], m.a.n.–mean atomic number (e^-).

	**ElB-1**	**ElB-2**	**ElKhB-1**	**ElKhB-2**
		**Si1**		
**BVS (vu)**	4.141	4.111	4.146	4.140
**ECoN**	3.9783	3.9781	3.9818	3.9827
**Vp (Å^3)**	2.139	2.151	2.139	2.137
**r_v (Å)**	1.610	1.613	1.610	1.610
**Δ_v (Å)**	0.045	0.044	0.042	0.040
**r_s (Å)**	1.611	1.613	1.610	1.610
**Δ (Å)**	0.033	0.033	0.031	0.030
**Vs (Å^3)**	17.499	17.592	17.494	17.480
**ECCv**	0.0605	0.0605	0.0559	0.0540
**SPHv**	0.9999	0.9999	0.9999	0.9998
		**Si2**		
**BVS (vu)**	4.089	4.088	4.114	4.122
**ECoN**	3.9737	3.9695	3.9646	3.9658
**Vp (Å^3)**	2.164	2.163	2.155	2.152
**r_v (Å)**	1.616	1.616	1.614	1.613
**Δ_v (Å)**	0.035	0.036	0.035	0.034
**r_s (Å)**	1.616	1.616	1.614	1.613
**Δ (Å)**	0.036	0.039	0.042	0.041
**Vs (Å^3)**	17.683	17.677	17.602	17.578
**ECCv**	0.0647	0.0703	0.0753	0.0743

**SPHv**	1	0.9999	0.9999	1
		**Si3**		
**BVS (vu)**	4.093	4.066	4.132	4.125
**ECoN**	3.9816	3.9821	3.9824	3.9862
**Vp (Å^3)**	2.158	2.167	2.145	2.146
**r_v (Å)**	1.615	1.617	1.611	1.612
**Δ_v (Å)**	0.051	0.050	0.048	0.045
**r_s (Å)**	1.615	1.617	1.611	1.612
**Δ (Å)**	0.031	0.031	0.031	0.027
**Vs (Å^3)**	17.647	17.720	17.529	17.543
**ECCv**	0.0565	0.0561	0.0557	0.0493
**SPHv**	0.9999	0.9999	0.9999	1
		**Zr**		
**m.a.n.**	41.4	41.44	40.928	40.96
**BVS (vu)**	4.026	3.986	3.988	4.004
**ECoN**	5.9937	5.9964	5.9909	5.9901
**Vp (Å^3)**	11.901	11.968	11.972	11.940
**r_v (Å)**	2.075	2.079	2.079	2.078
**Δ_v (Å)**	0.004	0.007	0.003	0.002
**r_s (Å)**	2.075	2.079	2.079	2.078
**Δ (Å)**	0.010	0.007	0.008	0.007
**Vs (Å^3)**	37.448	37.653	37.667	37.568
**ECCv**	0.0151	0.0095	0.0122	0.0105
**SPHv**	0.9846	0.9877	0.9797	0.9784
		**Na1**		
**m.a.n.**	11.154	10.89	8.525	7.546
**BVS (vu)**	0.937	0.922	0.938	0.947
**ECoN**	5.6296	5.6644	5.3716	5.1570
**Vp (Å^3)**	29.785	29.999	30.125	30.086
**r_v (Å)**	2.621	2.627	2.630	2.629
**Δ_v (Å)**	0.301	0.310	0.301	0.297
**r_s (Å)**	2.654	2.660	2.664	2.660
**Δ (Å)**	0.115	0.112	0.125	0.139
**Vs (Å^3)**	78.263	78.847	79.199	79.343
**ECCv**	0.1246	0.1210	0.1346	0.1488
**SPHv**	0.7652	0.7624	0.7473	0.7409
	**Na2**		**Na2/Ca2**	
**m.a.n.**	10.89	10.934	13.255	13.849
**BVS (vu)**	0.976	0.974	1.332	1.172
**ECoN**	5.5608	5.5542	5.6192	5.6055
**Vp (Å^3)**	18.711	18.744	18.533	18.361
**r_v (Å)**	2.469	2.471	2.460	2.452
**Δ_v (Å)**	0.050	0.052	0.035	0.028
**r_s (Å)**	2.469	2.471	2.460	2.452
**Δ (Å)**	0.049	0.051	0.034	0.027
**Vs (Å^3)**	63.022	63.173	62.340	61.782
**ECCv**	0.0583	0.0604	0.0406	0.0325
**SPHv**	0.8754	0.8748	0.8807	0.8790

Table 13. Calculated distortion parameters for polyhedra in the crystal structures of studied elpidite samples. υ-volume distortion [20,21], BLD-bond length distortion [22], ELD-edge length distortion [22], TAV-tetrahedral angle variance [23], TQE-tetrahedral quadratic elongation [23].

	ElB-1	ElB-2	ElKhB-1	ElKhB-2
		Si1		
υ	0.0023	0.0020	0.0019	0.0016
BLD (%)	1.0242	1.0070	0.9159	0.9001
ELD (%)	1.0944	0.9827	0.9762	1.0018
TAV	8.6541	7.4989	6.8270	6.6881
TQE	1.0022	1.0019	1.0017	1.0017
		Si2		
υ	0.0012	0.0011	0.0008	0.0020
BLD (%)	1.0867	1.1531	1.2633	1.2422
ELD (%)	0.8405	0.6780	0.6051	0.5674
TAV	3.5513	3.5247	2.4652	2.2345
TQE	1.0011	1.0011	1.0008	1.0008
		Si3		
υ	0.0018	0.0017	0.0015	0.0008
BLD (%)	1.0523	1.0507	1.0036	0.8885
ELD (%)	0.9797	0.9341	0.8235	0.8782
TAV	8.2738	7.6773	6.7584	6.8802
TQE	1.0020	1.0018	1.0016	1.0017
		Zr		
υ	0.0016	0.0014	0.0015	0.0017
BLD (%)	0.4711	0.3634	0.5878	0.6310
ELD (%)	1.4310	1.3472	1.3021	1.2831
OAV	3.5374	3.2871	3.2419	3.4911
OQE	1.0010	1.0009	1.0010	1.0011
		Na1		
υ	0.1220	0.1222	0.1225	0.1252
BLD (%)	6.9028	6.9225	7.5513	7.8716
ELD (%)	15.4579	15.4685	16.6580	15.8934
	Na2		NaCa2	
υ	0.0673	0.0679	0.0660	0.0664
BLD (%)	3.5372	3.5613	3.4061	3.4701
ELD (%)	7.9868	8.0625	7.8156	7.8037
OAV	155.0161	156.2597	152.4819	152.6757
OQE	1.0495	1.0500	1.0482	1.0484

Table 14. Valence balance calculation for studied elpidite sample (ElB-1).

	Na1	Na2	Zr	Si1	Si2	Si3	Σ
**O1**	-	-	-	1.039	-	1.045	**2.084**
**O2**	0.098	-	-	0.985[×2]	-	-	**2.068**
**O3**	-	0.150[×2]	0.673[×2]	1.120	-	-	**1.943**
**O4**	-	-	-	0.997	1.003	-	**2.000**
**O5**	0.174	-	-	-	0.999[×2]	-	**2.172**
**O6**	0.084[×2]	-	0.693[×2]	-	1.117	-	**1.894**
**O7**	-	-	-	-	0.970	0.975	**1.945**

**O8**	0.181	-	-	-	-	0.982(×2)	**2.145**
**O9**	0.056[×2]	0.122[×2]	0.647[×2]	-	-	1.091	**1.916**
**Ow1**	-	0.216[×2]	-	-	-	-	**0.204**
**Ow2**	0.204	-	-	-	-	-	**0.216**
**Σ**	**0.937**	**0.976**	**4.026**	**4.141**	**4.089**	**4.093**	

[×2] –valence strengths doubled in the calculation of valence balance at the cations
(×2)–valence strengths doubled in the calculation of valence balance at the anions
The values Ro and *B* for ion pairs involving oxygen were obtained in [14].

Table 15. Valence balance calculation for studied elpidite sample (ElB-2).

	Na1	Na2	Zr	Si1	Si2	Si3	Σ
**O1**	-	-	-	1.031	-	1.042	**2.073**
**O2**	0.097	-	-	0.982(×2)	-	-	**2.061**
**O3**	-	0.150[×2]	0.663[×2]	1.111	-	-	**1.924**
**O4**	-	-	-	0.987	1.008	-	**1.995**
**O5**	0.173	-	-	-	0.992[×2]	-	**2.157**
**O6**	0.083[×2]	-	0.683[×2]	-	1.123	-	**1.889**
**O7**	-	-	-	-	0.965	0.972	**1.937**
**O8**	0.180	-	-	-	-	0.972(×2)	**2.124**
**O9**	0.054[×2]	0.121[×2]	0.647[×2]	-	-	1.080	**1.902**
**Ow1**	-	0.216[×2]	-	-	-	-	**0.216**
**Ow2**	0.198	-	-	-	-	-	**0.198**
**Σ**	**0.922**	**0.974**	**3.986**	**4.111**	**4.088**	**4.066**	

[×2]–valence strengths doubled in the calculation of valence balance at the cations
(×2)–valence strengths doubled in the calculation of valence balance at the anions
The values Ro and *B* for ion pairs involving oxygen were obtained in [14].

Table 16. Valence balance calculation for studied elpidite sample (ElKhB-1).

	Na1	Na/Ca2*	Zr	Si1	Si2	Si3	Σ
**O1**	-	-	-	1.034	-	1.047	**2.081**
**O2**	0.092	-	-	0.992(×2)	-	-	**2.076**
**O3**	-	0.171[×2]	0.660[×2]	1.117	-	-	**1.948**
**O4**	-	-	-	1.003	0.997	-	**2.000**
**O5**	0.184	-	-	-	1.005 [×2]	-	**2.194**
**O6**	0.080[×2]	-	0.695[×2]	-	1.140	-	**1.915**
**O7**	-	-	-	-	0.972	0.987	**1.959**
**O8**	0.197	-	-	-	-	0.993(×2)	**2.183**
**O9**	0.053[×2]	0.147[×2]	0.639[×2]	-	-	1.105	**1.944**
**Ow1**	-	0.348[×2]	-	-	-	-	**0.348**
**Ow2**	0.199	-	-	-	-	-	**0.199**
**Σ**	**0.938**	**1.332**	**3.988**	**4.146**	**4.114**	**4.132**	

[×2]–valence strengths doubled in the calculation of valence balance at the cations
(×2)–valence strengths doubled in the calculation of valence balance at the anions
*calculated according refined occupancies
The values Ro and *B* for ion pairs involving oxygen were obtained in [14].

Table 17. Valence balance calculation for studied elpidite sample (ElKhB-2).

	Na1	Na/Ca2*	Zr	Si1	Si2	Si3	Σ
**O1**	-	-	-	1.037	-	1.045	**2.082**

**O2**	0.088	-	-	0.992(×2)	-	-	**2.072**
**O3**	-	0.173[×2]	0.660[×2]	1.111	-	-	**1.944**
**O4**	-	-	-	1.000	1.000	-	**2.000**
**O5**	0.186	-	-	-	1.010 [×2]	-	**2.206**
**O6**	0.080[×2]	-	0.700[×2]	-	1.140	-	**1.920**
**O7**	-	-	-	-	0.972	0.992	**1.964**
**O8**	0.203	-	-	-	-	0.994(×2)	**2.191**
**O9**	0.052[×2]	0.154[×2]	0.642[×2]	-	-	1.094	**1.942**
**Ow1**	-	0.259[×2]	-	-	-	-	**0.259**
**Ow2**	0.206	-	-	-	-	-	**0.206**
**Σ**	**0.947**	**1.172**	**4.004**	**4.140**	**4.122**	**4.125**	

[×2]–valence strengths doubled in the calculation of valence balance at the cations
(×2)–valence strengths doubled in the calculation of valence balance at the anions
*calculated according refined occupancies
The values R_0 and *B* for ion pairs involving oxygen were obtained in [14].

Table 18. Crystallographic coordinates of the modeled elpidites. The crystal structures were simulated in the space group *P*1. Position occupancies are taken as 1.

**Site**	**$Na_2ZrSi_6O_{15}$·$3H_2O$ model**			**$Na_{1.5}Ca_{0.25}ZrSi_6O_{15}$·$2.75H_2O$ model**			**$CaZrSi_6O_{15}$·$2H_2O$ model**		
	x/a	*y/b*	*z/c*	*x/a*	*y/b*	*z/c*	*x/a*	*y/b*	*z/c*
Zr-1	0.4940	0.2507	0.5000	0.4959	0.2516	0.4997	0.5000	0.2500	0.5000
Zr-2	0.5060	0.7507	0.5000	0.5045	0.7504	0.5003	0.5000	0.7500	0.5000
Zr-3	0.5060	0.7507	0.0000	0.5053	0.7496	-0.0003	0.5000	0.7500	0.0000
Zr-4	0.4940	0.2507	0.0000	0.4937	0.2517	0.0022	0.5000	0.2500	0.0000
Si1-1	0.7696	0.3865	0.6462	0.7707	0.3914	0.6430	0.7748	0.3881	0.6462
Si1-2	0.2304	0.6150	0.3539	0.2294	0.6099	0.3564	0.2252	0.6119	0.3538
Si1-3	0.2304	0.6149	0.1461	0.2305	0.6186	0.1499	0.2252	0.6119	0.1462
Si1-4	0.7696	0.3866	0.8540	0.7676	0.3801	0.8503	0.7748	0.3881	0.8538
Si1-5	0.2304	0.8866	0.8540	0.2329	0.8815	0.8509	0.2252	0.8881	0.8538
Si1-6	0.7696	0.1149	0.1461	0.7695	0.1169	0.1471	0.7748	0.1119	0.1462
Si1-7	0.7696	0.1150	0.3539	0.7699	0.1079	0.3571	0.7748	0.1119	0.3538
Si1-8	0.2304	0.8864	0.6462	0.2298	0.8932	0.6420	0.2252	0.8881	0.6462
Si2-1	0.5108	0.0488	0.6409	0.5082	0.0549	0.6464	0.5000	0.0477	0.6409
Si2-2	0.4892	0.9526	0.3592	0.4944	0.9480	0.3553	0.5000	0.9523	0.3591
Si2-3	0.4892	0.9525	0.1410	0.4926	0.9576	0.1353	0.5000	0.9523	0.1409
Si2-4	0.5108	0.0489	0.8591	0.5108	0.0439	0.8657	0.5000	0.0477	0.8591
Si2-5	0.4892	0.5489	0.8591	0.4903	0.5421	0.8643	0.5000	0.5477	0.8591
Si2-6	0.5108	0.4525	0.1410	0.5112	0.4559	0.1368	0.5000	0.4523	0.1409
Si2-7	0.5108	0.4526	0.3592	0.5091	0.4470	0.3525	0.5000	0.4523	0.3591
Si2-8	0.4892	0.5488	0.6409	0.4924	0.5526	0.6447	0.5000	0.5477	0.6409
Si3-1	0.2184	0.3944	0.6441	0.2204	0.3971	0.6429	0.2252	0.3881	0.6461
Si3-2	0.7816	0.6070	0.3560	0.7793	0.6048	0.3578	0.7748	0.6119	0.3539
Si3-3	0.7816	0.6069	0.1440	0.7814	0.6124	0.1468	0.7748	0.6119	0.1461
Si3-4	0.2184	0.3945	0.8560	0.2158	0.3896	0.8536	0.2252	0.3881	0.8539
Si3-5	0.7816	0.8945	0.8560	0.7842	0.8908	0.8542	0.7748	0.8881	0.8539
Si3-6	0.2184	0.1069	0.1440	0.2188	0.1141	0.1463	0.2252	0.1119	0.1461
Si3-7	0.2184	0.1070	0.3560	0.2215	0.1053	0.3577	0.2252	0.1119	0.3539
Si3-8	0.7816	0.8944	0.6441	0.7800	0.8982	0.6424	0.7748	0.8881	0.6461
O1-1	0.9931	0.4064	0.6422	0.9948	0.4096	0.6413	0.0000	0.4024	0.6441

O1-2	0.0069	0.5950	0.3579	0.0048	0.5921	0.3600	0.0000	0.5976	0.3559
O1-3	0.0069	0.5949	0.1421	0.0065	0.6016	0.1444	0.0000	0.5976	0.1441
O1-4	0.9931	0.4065	0.8579	0.9907	0.3998	0.8490	0.0000	0.4024	0.8559
O1-5	0.0069	0.9065	0.8579	0.0096	0.9012	0.8565	0.0000	0.9024	0.8559
O1-6	0.9931	0.0949	0.1421	0.9938	0.0994	0.1435	0.0000	0.0976	0.1441
O1-7	0.9931	0.0950	0.3579	0.9955	0.0937	0.3653	0.0000	0.0979	0.3559
O1-8	0.0069	0.9064	0.6422	0.0054	0.9123	0.6399	0.0000	0.9024	0.6441
O2-1	0.7079	0.3555	0.7501	0.7010	0.3581	0.7440	0.7076	0.3649	0.7500
O2-2	0.2921	0.6459	0.2500	0.2899	0.6482	0.2548	0.2924	0.6351	0.2500
O2-3	0.2921	0.8555	0.7501	0.2944	0.8597	0.7445	0.2924	0.8649	0.7500
O2-4	0.7079	0.1459	0.2500	0.7092	0.1350	0.2538	0.7076	0.1351	0.2500
O3-1	0.7102	0.3094	0.5755	0.7242	0.3133	0.5691	0.7229	0.3071	0.5764
O3-2	0.2898	0.6920	0.4246	0.2917	0.6811	0.4328	0.2771	0.6929	0.4236
O3-3	0.2898	0.6918	0.0753	0.2928	0.6977	0.0816	0.2771	0.6929	0.0764
O3-4	0.7102	0.3097	0.9248	0.7077	0.2961	0.9122	0.7229	0.3071	0.9236
O3-5	0.2898	0.8097	0.9248	0.2924	0.7979	0.9139	0.2771	0.8071	0.9236
O3-6	0.7102	0.1918	0.0753	0.7090	0.1981	0.0815	0.7229	0.1929	0.0764
O3-7	0.7102	0.1920	0.4246	0.7163	0.1856	0.4305	0.7229	0.1929	0.4236
O3-8	0.2898	0.8094	0.5755	0.2896	0.8192	0.5682	0.2771	0.8071	0.5764
O4-1	0.6756	0.4859	0.6233	0.6771	0.4903	0.6187	0.6881	0.4871	0.6153
O4-2	0.3244	0.5155	0.3768	0.3187	0.5078	0.3709	0.3119	0.5129	0.3847
O4-3	0.3243	0.5153	0.1234	0.3236	0.5200	0.1257	0.3119	0.5129	0.1153
O4-4	0.6757	0.4861	0.8767	0.6728	0.4769	0.8822	0.6881	0.4871	0.8847
O4-5	0.3243	0.9861	0.8767	0.3295	0.9776	0.8827	0.3119	0.9871	0.8847
O4-6	0.6757	0.0153	0.1234	0.6799	0.0187	0.1144	0.6881	0.0129	0.1153
O4-7	0.6756	0.0155	0.3768	0.6802	0.0084	0.3837	0.6881	0.0129	0.3847
O4-8	0.3244	0.9859	0.6233	0.3195	0.9950	0.6218	0.3119	0.9871	0.6153
O5-1	0.5326	0.0695	0.7500	0.5242	0.0681	0.7565	0.5001	0.0667	0.7500
O5-2	0.4674	0.9319	0.2501	0.4840	0.9360	0.2448	0.4999	0.9333	0.2500
O5-3	0.4674	0.5695	0.7500	0.4781	0.5655	0.7551	0.4999	0.5667	0.7500
O5-4	0.5326	0.4319	0.2501	0.5305	0.4272	0.2440	0.5001	0.4333	0.2500
O6-1	0.4886	0.1438	0.5909	0.4964	0.1531	0.6012	0.4999	0.1383	0.5821
O6-2	0.5114	0.8576	0.4092	0.5036	0.8493	0.3987	0.5001	0.8617	0.4179
O6-3	0.5113	0.8575	0.0911	0.5018	0.8639	0.0817	0.5001	0.8617	0.0821
O6-4	0.4887	0.1440	0.9090	0.4865	0.1360	0.9198	0.4999	0.1383	0.9179
O6-5	0.5113	0.6440	0.9089	0.5137	0.6353	0.9181	0.5001	0.6383	0.9179
O6-6	0.4887	0.3575	0.0911	0.4939	0.3709	0.0709	0.4999	0.3617	0.0821
O6-7	0.4886	0.3576	0.4092	0.4927	0.3555	0.4103	0.4999	0.3617	0.4179
O6-8	0.5113	0.6438	0.5909	0.5077	0.6513	0.6016	0.5001	0.6383	0.5821
O7-1	0.3001	0.4934	0.6074	0.3051	0.4962	0.6094	0.3118	0.4871	0.6152
O7-2	0.6999	0.5080	0.3927	0.6960	0.5040	0.3877	0.6882	0.5129	0.3848
O7-3	0.6999	0.5079	0.1074	0.6990	0.5153	0.1085	0.6882	0.5129	0.1152
O7-4	0.3001	0.4935	0.8926	0.2951	0.4884	0.8916	0.3118	0.4871	0.8848
O7-5	0.6999	0.9935	0.8926	0.7057	0.9895	0.8926	0.6882	0.9871	0.8848
O7-6	0.3001	0.0079	0.1074	0.3024	0.0155	0.1098	0.3118	0.0129	0.1152
O7-7	0.3001	0.0080	0.3927	0.3072	0.0057	0.3882	0.3118	0.0129	0.3848
O7-8	0.6999	0.9934	0.6074	0.6973	0.9995	0.6118	0.6882	0.9871	0.6152
O8-1	0.2919	0.3829	0.7501	0.2943	0.3796	0.7472	0.2924	0.3650	0.7500
O8-2	0.7081	0.6186	0.2500	0.7072	0.6235	0.2528	0.7076	0.6350	0.2500

O8-3	0.7081	0.8829	0.7501	0.7103	0.8807	0.7480	0.7076	0.8650	0.7500
O8-4	0.2919	0.1186	0.2501	0.2837	0.1277	0.2533	0.2924	0.1350	0.2500
O9-1	0.2894	0.3110	0.5834	0.2770	0.3143	0.5759	0.2771	0.3071	0.5764
O9-2	0.7106	0.6904	0.4166	0.7121	0.6842	0.4248	0.7229	0.6929	0.4236
O9-3	0.7107	0.6903	0.0833	0.7103	0.6973	0.0875	0.7229	0.6929	0.0764
O9-4	0.2893	0.3112	0.9168	0.2895	0.3046	0.9109	0.2771	0.3071	0.9236
O9-5	0.7107	0.8112	0.9168	0.7094	0.8067	0.9133	0.7229	0.8081	0.9236
O9-6	0.2893	0.1903	0.0833	0.2825	0.1968	0.0832	0.2771	0.1929	0.0764
O9-7	0.2894	0.1904	0.4166	0.2786	0.1846	0.4291	0.2771	0.1929	0.4236
O9-8	0.7106	0.8110	0.5834	0.7110	0.8188	0.5762	0.7229	0.8071	0.5764
Na1-1	0.4407	0.2335	0.7499	0.4505	0.2321	0.7705			
Na1-2	0.5593	0.7679	0.2501	0.5531	0.7698	0.2382			
Na1-3	0.5593	0.7335	0.7499	0.5552	0.7317	0.7627			
Na1-4	0.4407	0.2679	0.2501						
Na2-1	0.0006	0.2507	0.5000						
Ca2-1				−0.0014	0.2506	0.4987	0.0000	0.2500	0.5000
Na2-2	0.9994	0.7507	0.5000	0.0005	0.7500	0.4999			
Ca2-2							0.0000	0.7500	0.5000
Na2-3	0.9994	0.7507	0.0000	0.0011	0.7497	0.0001			
Ca2-3							0.0000	0.7500	0.0000
Na2-4	0.0006	0.2507	0.0000	−0.0022	0.2521	−0.0001			
Ca2-4							0.0000	0.2500	0.0000
Ow1-1	0.0182	0.1113	0.5884	0.0096	0.1142	0.5884	0.0001	0.1058	0.5702
H1-1	0.9058	0.0719	0.5915	0.8971	0.0742	0.5938	−0.1108	0.0672	0.5828
H2-1	0.1262	0.0692	0.5886	0.1200	0.0730	0.5903	0.1111	0.0672	0.5828
Ow1-2	0.9818	0.8910	0.4117	0.9888	0.8857	0.4075	−0.0001	0.8942	0.4298
H1-2	0.0942	0.9296	0.4086	0.1017	0.9247	0.4057	0.1108	0.9328	0.4172
H2-2	0.8738	0.9323	0.4114	0.8810	0.9279	0.4104	0.8889	0.9328	0.4172
Ow1-3	0.9818	0.8900	0.0885	0.9858	0.8953	0.0823	−0.0001	0.8942	0.0702
H1-3	0.0943	0.9295	0.0915	0.0976	0.9348	0.0883	0.1108	0.9328	0.0828
H2-3	0.8738	0.9322	0.0887	0.8779	0.9377	0.0819	0.8889	0.9328	0.0828
Ow1-4	0.0182	0.1114	0.9116	0.0207	0.1078	0.9154	0.0001	0.1058	0.9298
H1-4	0.9057	0.0720	0.9085	0.9093	0.0678	0.9107	−0.1108	0.0672	0.9172
H2-4	0.1262	0.0693	0.9113	0.1292	0.0664	0.9199	0.1111	0.0672	0.9172
Ow1-5	0.9818	0.6114	0.9116	0.9789	0.6052	0.9180	−0.0001	0.6058	0.9298
H1-5	0.0943	0.5720	0.9085	0.0910	0.5655	0.9140	0.1108	0.5672	0.9172
H2-5	0.8738	0.5693	0.9113	0.8717	0.5629	0.9211	0.8889	0.5672	0.9172
Ow1-6	0.0182	0.3900	0.0885	0.0122	0.4040	0.0529	0.0001	0.3942	0.0702
H1-6	0.9057	0.4295	0.0915	0.9034	0.4410	0.0717	−0.1108	0.4328	0.0828
H2-6	0.1262	0.4322	0.0887	0.1232	0.4373	0.0746	0.1111	0.4328	0.0828
Ow1-7	0.0182	0.3901	0.4117	0.0077	0.3893	0.4231	0.0001	0.3942	0.4298
H1-7	0.9058	0.4296	0.4086	0.8967	0.4284	0.4116	−0.1108	0.4328	0.4172
H2-7	0.1262	0.4323	0.4114	0.1189	0.4259	0.4064	0.1111	0.4328	0.4172
Ow1-8	0.9818	0.6113	0.5884	0.9864	0.6157	0.5938	−0.0001	0.6058	0.5702
H1-8	0.0942	0.5719	0.5915	0.0990	0.5764	0.5942	0.1108	0.5672	0.5828
H2-8	0.8738	0.5692	0.5886	0.8786	0.5736	0.5903	0.8889	0.5672	0.5828
Ow2-1	0.1215	0.1954	0.7500	0.1275	0.1913	0.7560			
H3-1	0.0707	0.1639	0.6951	0.0713	0.1603	0.7028			
H4-1	0.0707	0.1639	0.8049	0.0777	0.1608	0.8121			

Ow2-2	0.8785	0.8060	0.2501	0.8733	0.8111	0.2435
H3-2	0.9293	0.8375	0.3050	0.9298	0.8392	0.2988
H4-2	0.9294	0.8375	0.1952	0.9268	0.8422	0.1890
Ow2-3	0.8785	0.6954	0.7500	0.8758	0.6921	0.7578
H3-3	0.9294	0.6640	0.8049	0.9244	0.6588	0.8122
H4-3	0.9293	0.6639	0.6951	0.9297	0.6628	0.7024
Ow2-4	0.1215	0.3060	0.2501			
H3-4	0.0706	0.3375	0.1952			
H4-4	0.0707	0.3375	0.3050			

Table 19. The positions of the bands (cm^{-1}) in the IR spectra of elpidite from literature.

	ElMSHil	ElLov(a)	ElKhB(b)	ElLov(c) ElKhB(d)	ElLov(e)	ElKhib	ElLov(f)	ElKhB(g)
ν(H-O-H)	3533, 3506, 3454	3540, 3490, 3435	3551, 3505, 3453	3551, 3506, 3453, 3250				
δ(H-O-H)	1638	1660, 1640, 1620	1647, 1638	1639				
ν(Si-O-Si)	1200-1100							
ν(Si-O)			1167, 1117,	1170–1115				
δ(Si-O-Si)	800-500		1011, 495,	below 550				
δ(Si-O-Zr)			434	1050-1010				
ν(Zr-O)			642, 629	700-600				
δ(O-Si-O)				810-770				
Not attributed		1168, 1117, 1050, 1035, 1010, 935, 808, 779, 708, 674, 646, 626, 597, 543, 520, 490, 450, 430,	809, 779, 737, 710		3550, 3505, 3450, 3245w, 1645, 1168s, 1114s, 1050sh, 1031s, 1009s, 808w, 778, 741w, 709, 645, 626, 599, 542, 522, 490sh, 460sh, 450sh, 430	3555, 3445, 3260sh, 1655sh, 1637, 1165sh, 1124s, 1018s, 802w, 780, 706w, 642, 630sh, 600sh, 535sh, 510sh, 450sh, 431s	3550, 3505, 3450, 3235w, 1640, 1169s, 1117s, 1050sh, 1034s, 1010s, 808w, 778, 737w, 708w, 680sh, 646, 626, 596, 542, 520w, 491w, 465sh, 450sh, 429s	3545, 3500sh, 3445, 3250w, 1640, 1166s, 1116s, 1030sh, 1020sh, 1012s, 809w, 780, 710w, 642, 627, 596, 541, 500sh, 450sh, 423s

Note: ν–stretching vibrations, δ–bending vibrations, w–weak band, sh–shoulder.
ElMSHil(a)–elpidite from Mt St. Hilaire (Canada) [9]; ElLov(a)–elpidite from Mount Alluav, Lovozero (Russia) [6]; ElKhB(b)–elpidite from Khan-Bogdo (Mongolia) [7]; ElLov(c)–elpidite from Mount Alluav, Lovozero (Russia) and ElKhB(d)–elpidite from Khan-Bogdo (Mongolia) [11]; ElLov(e)–elpidite from Kedykverpakhk, Lovozero (Russia) [24]; ElKhi–elpidite from Yukspor, Khibiny (Russia) [24]; ElLov(f)–elpidite from Umbozero mine, Lovozero (Russia) [24]; ElKhB(g)–elpidite from Khan-Bogdo (Mongolia) [24].

Table 20. Calculated vibrational modes in simulated structure models of elpidite (framework–Zr+Si1+S2+Si3).

$Na_2ZrSi_6O_{15}$·$3H_2O$ model			$Na_{1.5}Ca_{0.25}ZrSi_6O_{15}$·$2.75H_2O$ model			$CaZrSi_6O_{15}$·$2H_2O$ model		
**Wavenumber (cm^{-1})**	**e^2/amu**	**Peak attribution**	**Wavenumber (cm^{-1})**	**e^2/amu**	**Peak attribution**	**Wavenumber (cm^{-1})**	**e^2/amu**	**Peak attribution**
96	0.49	Na1						
			112	0.1	Na1+ Na2/Ca2+W1			
129	0.16	Na2	128	0.05	Na1+ Na2/Ca2			
			129	0.04	Na1+ Na2/Ca2+Si1+Si3+W1			
			138	0.09	Na1+ Na2/Ca2+framework+W1+W2			
			142	0.06	Na1+ Na2/Ca2+Si1+Si3+W1+W2			
						153-159	0.31–0.82	Ca+framework+W1
			159–162	0.12–0.23	Na1+ Na2/Ca2+framework+W1+W2			
166	0.38	framework+W1+W2	164	0.07	framework+W1+W2			
			168	0.09	Si1+Si3+W1			
183	0.19	Na1+Na2+framework+W1+W2	174	0.08	Na1+ Na2/Ca2+framework+W1+W2			
195	0.16	Na2+framework+W1	191	0.05	Na2/Ca2+framework+W1			
			196-216	0.07-0.10	Na1+Na2/Ca2+framework+W1+W2			
						200	0.33	Ca+framework+W1
						225	0.7	Zr+Si1+Si3
			224	0.20	Na1+Na2/Ca2+Si1+Si3+W1+W2			
226	0.28	Na2+Si1+W1+W2						
			228	0.04	Na1+framework+W1			
232	0.22	Na2+Zr+Si1+Si3+W1+W2	229	0.16	Na1+ Na2/Ca2+Zr+Si1+Si3+W1+W2			
			231	0.13	Na2/Ca2+framework +W1+W2			
						240	2.35	framework
241	0.24	Na1+Na2+framework+W1						
			237	0.04	Si1+Si3+W1+W2			
			243	0.07	Na1+Si1+Si3+W1+W2			
						243	0.20	Ca+Si1+Si2+Si3

			245	0.09	Na1+ Na2/Ca2+framework+W1+W2			
			246	0.32	Na1+ Na2/Ca2+Si1+Si2+Si3+W1+W2			
246-251	0.58-1.82	Na1 + Na2 + framework + W1 + W2	249-253	0.09-0.99	Na1+ Na2/Ca2+framework+W1+W2			
			254	0.06	W1+W2			
						258-260	0.77-0.82	framework
259	0.64	Na2+framework+W1+W2	260	0.55	Na2/Ca2+framework+W1+W2			
270	0.12	Si1+Si2+Si3+W1+W2						
271	0.62	Na1+Na2+framework+W1+W2	263–272	0.06–0.42	Na1+ Na2/Ca2+framework+W1+W2			
273	0.87	Na2+Zr+Si2+W1+W2						
			275-283	0.05–0.26	framework+W1+W2			
			284	0.09	Na1+framework+W1+W2			
281	0.24	Si1+Si2+Si3						
			285–288	0.07–0.08	framework+W1+W2			
						286	0.27	framework +W1
			289	0.12	Na1+framework+W1+W2			
290	0.23	Si1+Si2+Si3+W1						
			293	0.05	framework+W1			
			297	0.10	framework+W1+W2			
305	0.18	framework+W1	304	0.09	framework+W1			
311	0.14	Si1+Si2+Si3+W1						
			307–310	0.05–0.24	framework +W1+W2			
312	1.04	framework				318	1.3	framework
316	0.46	framework+W1	310-328	0.11–0.42	framework+W1			
			355	0.06	Na2/Ca2+ framework +W1	323	0.30	Ca+framework +W1
						337	0.24	Ca+W1
396	0.81	Si1+Si2+Si3						
						399	0.65	Ca+framework+W1

			398–405	0.24–0.67	framework+W1			
401–420	1.20-3.90	Si1+Si2+Si3+W1						
			406–409	1.24–2.69	Si1+Si2+Si3+W1+W2			
			410–422	0.05–0.58	framework +W1	411	6.61	framework+W1
			470	0.08	Si1+Si2+Si3+W1			
			475–478	0.14–0.18	W1			
			478	0.12	Si2+Si3+W1+W2			
			480	0.16	W1			
480	1.08	framework+W1+W2	482	0.63	framework+W1+W2			
			484	0.16	Si1+Si2+Si3+W1+W2			
						481	0.64	Si1+Si3+W1
487	0.44	Si1+W1						
						489	0.79	Ca+framework+W1
			495	0.17	W1			
			498	0.07	Si1+W1+W2			
			501	0.39	W1+W2			
			507-513	0.08-0.16	W1			
			518	0.17	W1+W2			
505	0.26	Si1+Si2+Si3+W1+W2	520	0.30	Si1+Si2+Si3+W1+W2			
						512	0.59	Si1+Si2+Si3+W1
						513	0.55	framework+W1
522	0.83	Si1+Si2+Si3+W1						
			526-530	0.14–0.22	W1+W2			
			535	0.06	W1			
547–574	0.17–1.43	W1+W2	537–580	0.06–0.35	W1+W2			
			580–586	0.04–0.16	W1	561-597	0.21-2.83	W1
588–609	0.18-1.24	framework+W1+W2						
609	0.17	W1+W2	602–607	0.05–0.28	W1+W2			
			607	0.29	Si1+Si2+Si3+W1			
						615	1.91	framework+W1
616	0.30	Si2+Si3+W1+W2						
623	2.62	W1+W2	612–633	0.11–1.68	W1+W2			
						632	1.75	framework

			635	0.18	Si1+Si3+W1+W2			
636	0.38	Si1+W1+W2						
637	0.35	Si1+Si2+Si3+W1+W2						
			636–664	0.06–0.21	W1	652	0.56	W1
			688	0.08	Si1+Si3+W1+W2			
			691–696	0.08–0.13	Si1+Si2+Si3+W1+W2			
691	0.17	framework+W1+W2						
						694-768	0.48-2.00	framework
			708	0.18	W1+W2			
720	0.88	Si1+Si2+Si3+W1+W2	716–718	0.12–0.13	Si1+Si2+Si3+W1+W2			
735	0.79	W1+W2	735–743	0.05–0.33	W1+W2			
740-792	0.14-1.18	Si1+Si2+Si3+W1+W2	748	0.12	Si1+Si2+Si3+W1+W2			
			760	0.04	W1+W2			
			761	1.27	Si1+Si2+Si3+W1+W2			
			763	0.13	Si2+Si3+W1+W2			
			765	0.11	W1			
			790-793	0.23–0.52	Si1+Si3+W1+W2			
						801	0.67	Si1 + Si2 + Si3
861	0.55	W1+W2	833-888	0.09–0.23	W1+W2			
			948	0.81	Si1+Si3			
						950–980	3.68-5.80	framework
			953	1.56	Si1+Si2+Si3			
			954	0.26	Si2+Si3			
			960	2.73	Si1+Si3			
976	3.04	Si2+Si3	963-966	0.05-0.70	Si2+Si3			
			973-978	0.28-2.45	Si1+Si2+Si3			
			981	2.43	Si1+Si3			
982	7.3	framework	985-998	0.62-3.15	framework	984-1005	2.19-18.88	framework
986-999	1.26-5.02	Zr+Si1+Si3	1000	0.61	Zr+Si1+Si3			
1010	10.04	framework	1000-1029	0.4-3.74	framework			
1015	4.71	Zr+Si1+Si3						
1021-1022	3.35-7.07	Si1+Si2+Si3						
1030	7.51	framework						

			1031	1.35	Si1+Si2	1032	6.24	Si1+Si2
1074-1084	1.07-1.69	framework+W1	1054-1094	0.10-0.78	framework+W1	1061	2.68	framework+W1
						1085	2.27	Si1+Si3
			1097	0.08	Si1+Si2+Si3+W1+W2			
1100	0.20	Si1+Si2+Si3+W1	1111	0.11	Si1+Si2+Si3+W1			
1122	2.09	Si2	1120-1128	0.07-2.5	Si2			
			1142	0.12	Si1+Si3			
			1145-1148	0.06-0.12	Si1+Si2+Si3	1139	4.69	Si1+Si2+Si3
			1149	0.07	Si1+Si3			
			1152-1165	0.05-0.43	framework			
			1167-1170	0.38-0.61	Si1+Si3	1166	3.9	Si1+Si3
			1173	0.47	Si1+Si2+Si3			
			1175	0.26	Si1+Si3			
1171-1182	1.01-2.95	framework	1178-1188	0.21-1.70	framework			
						1592	1.45	W1
1589	1.26	W1+W2	1584-1589	0.04-0.72	W1+W2			
1592	0.14	W2	1591	0.07	W2			
1594	0.25	W1+W2	1597	0.17	W1+W2			
			1599	0.24	W1			
			1604	0.04	W1+W2			
3312-3347	0.85-4.97	W2	3262-3308	1.15-1.28	W2			
			3315	1.28	W1+W2			
			3327-3367	0.86-1.50	W2			
3459-3465	1.63-2.45	W1+W2	3385-3467	0.60-1.49	W1+W2			
						3420-3424	1.11-4.68	W1
			3473	1.05	W1			
						3471	10.09	W1
			3482-3487	0.21-0.89	W1+W2			
3517-3519	0.33-5.05	W1	3491-3517	0.68-1.20	W1			
			3519	1.01	W2			
			3532-3605	0.74-1.05	W1			

Table 21. Comparative geometrical parameters for tetrahedra and polyhedra in the crystal structures of studied samples and simulated models of $Na_2ZrSi_6O_{15}·3H_2O$ and $Na_{1.5}Ca_{0.25}ZrSi_6O_{15}·2.75H_2O$ elpidite. ABL–average bond length; P_{vol}–polyhedral volume; BLD-bond length distortion [22], TAV-tetrahedral angle variance [23], TQE-tetrahedral quadratic elongation [23].

Atom	ABL	P_{vol}	BLD	TQE/OQE	TAV/OAV	ABL	P_{vol}	BLD	TQE/OQE	TAV/OAV	Atom	ABL	P_{vol}	BLD	TQE/OQE	TAV/OAV
			ElB-1					ElB-2						$Na_2ZrSi_6O_{15}·3H_2O$ model		
											Zr-1	2.078	11.915	0.662	1.0023	7.9051
											Zr-2	2.078	11.915	0.661	1.0023	7.9053
											Zr-3	2.078	11.915	0.656	1.0023	7.9093
Zr	2.075	11.901	0.471	1.0010	3.5374	2.079	11.968	0.363	1.0009	3.2871	Zr-4	2.078	11.915	0.656	1.0023	7.9092
											Si1-1	1.624	2.187	1.211	1.0029	11.1793
											Si1-2	1.624	2.187	1.211	1.0029	11.1784
											Si1-3	1.624	2.188	1.213	1.0029	11.1797
											Si1-4	1.624	2.188	1.213	1.0029	11.1808
											Si1-5	1.624	2.188	1.213	1.0029	11.1797
											Si1-6	1.624	2.188	1.213	1.0029	11.1813
											Si1-7	1.624	2.187	1.211	1.0029	11.1787
Si1	1.611	2.139	1.024	1.0022	8.6541	1.614	2.151	1.007	1.0019	7.4989	Si1-8	1.624	2.187	1.211	1.0029	11.1793
											Si2-1	1.624	2.196	1.271	1.0012	3.4065
											Si2-2	1.624	2.196	1.271	1.0012	3.4062
											Si2-3	1.624	2.196	1.272	1.0012	3.4300
											Si2-4	1.624	2.196	1.272	1.0012	3.4297
											Si2-5	1.624	2.196	1.272	1.0012	3.4298
											Si2-6	1.624	2.196	1.272	1.0012	3.4291
											Si2-7	1.624	2.196	1.271	1.0012	3.4051
Si2	1.616	2.164	1.087	1.0011	3.5513	1.616	2.163	1.153	1.0011	3.5247	Si2-8	1.624	2.196	1.272	1.0012	3.4053
											Si3-1	1.627	2.200	1.377	1.0028	11.4875
											Si3-2	1.627	2.200	1.377	1.0028	11.4863
											Si3-3	1.627	2.200	1.378	1.0028	11.4831
											Si3-4	1.627	2.200	1.378	1.0028	11.4846
											Si3-5	1.627	2.200	1.378	1.0028	11.4837
											Si3-6	1.627	2.200	1.378	1.0028	11.4838
											Si3-7	1.627	2.200	1.377	1.0028	11.4868
Si3	1.616	2.158	1.052	1.0020	8.2738	1.618	2.167	1.051	1.0018	7.6773	Si3-8	1.627	2.200	1.377	1.0028	11.4863

											Na1-1	2.633	29.195	6.111		
											Na1-2	2.633	29.196	6.111		
											Na1-3	2.633	29.195	6.111		
Na1	2.645	29.785	6.903			2.652	29.999	6.923			Na1-4	2.633	29.195	6.111		
											Na2-1	2.490	19.267	1.740	1.0453	151.6649
											Na2-2	2.490	19.267	1.740	1.0453	151.6684
											Na2-3	2.490	19.257	1.743	1.0454	151.9255
Na2	2.469	18.711	3.537	1.0495	155.0161	2.471	18.744	3.561	1.0500	156.2597	Na2-4	2.490	19.257	1.743	1.0454	151.9252

**Atom**	**ABL**	**P_{vol}**	**BLD**	**TQE/ OQE**	**TAV/ OAV**	**ABL**	**P_{vol}**	**BLD**	**TQE/ OQE**	**TAV/ OAV**	**Atom**	**ABL**	**P_{vol}**	**BLD**	**TQE/ OQE**	**TAV/ OAV**
**ElKhB-1**						**ElKhB-2**					**$Na_{1.5}Ca_{0.25}ZrSi_6O_{15}$·$2.75H_2O$ model**					
											Zr-1	2.089	12.050	1.530	1.0061	19.0793
											Zr-2	2.082	12.015	0.316	1.0014	4.8155
											Zr-3	2.080	11.958	0.647	1.0020	6.7654
Zr	2.079	11.972	0.588	1.0010	3.2419	2.078	11.940	0.631	1.0011	3.4911	Zr-4	2.082	11.974	1.188	1.0031	9.7997
											Si1-1	1.624	2.191	0.668	1.0026	9.7587
											Si1-2	1.625	2.194	1.161	1.0030	11.8268
											Si1-3	1.623	2.186	1.078	1.0023	8.7445
											Si1-4	1.625	2.191	1.428	1.0034	14.2967
											Si1-5	1.623	2.188	1.141	1.0024	9.3672
											Si1-6	1.624	2.188	1.216	1.0029	11.6840
											Si1-7	1.624	2.188	1.216	1.0029	11.6840
Si1	1.611	2.139	0.916	1.0017	6.8270	1.611	2.137	0.900	1.0017	6.6881	Si1-8	1.628	2.199	1.311	1.0034	13.2361
											Si2-1	1.628	2.211	1.288	1.0009	2.0664
											Si2-2	1.626	2.204	1.375	1.0015	4.9548
											Si2-3	1.624	2.195	1.189	1.0010	3.1491
											Si2-4	1.625	2.197	1.450	1.0014	4.5107
											Si2-5	1.626	2.202	1.213	1.0013	3.9767
											Si2-6	1.625	2.200	1.337	1.0009	2.9911
											Si2-7	1.627	2.210	1.393	1.0007	1.8161
Si2	1.614	2.155	1.263	1.0008	2.4652	1.613	2.152	1.242	1.0008	2.2345	Si2-8	1.628	2.208	1.345	1.0018	5.7950
											Si3-1	1.628	2.206	0.790	1.0023	8.9086
											Si3-2	1.626	2.200	1.243	1.0026	10.5492
											Si3-3	1.624	2.193	1.157	1.0021	8.4697

											Si3-4	1.629	2.208	1.662	1.0036	15.5307
											Si3-5	1.627	2.201	1.365	1.0028	11.6244
											Si3-6	1.626	2.197	1.365	1.0030	12.0822
											Si3-7	1.627	2.199	0.635	1.0027	10.4764
Si3	1.612	2.145	1.004	1.0016	6.7584	1.612	2.146	0.889	1.0017	6.8802	Si3-8	1.628	2.206	1.391	1.0034	13.8750
											Na1-1	2.597	27.485	4.718		
											Na1-2	2.558	23.029	4.435		
Na1	2.654	30.125	7.551			2.653	30.086	7.872			Na1-3	2.574	23.345	4.414		
											Ca2-1	2.408	17.186	1.625	1.0551	170.7395
											Na2-2	2.483	18.909	2.021	1.0525	166.6039
											Na2-3	2.511	19.833	1.613	1.0425	144.5774
Na2/Ca2	2.460	18.533	3.406	1.0482	152.4819	2.453	18.361	3.470	1.0484	152.6757	Na2-4	2.485	19.046	1.959	1.0498	159.9986

Table 22. Calculated geometrical parameters for tetrahedra and polyhedra in the crystal structure of the simulated model of hypothetical $CaZrSi_6O_{15}\cdot 2H_2O$ elpidite. ABL–average bond length; P_{vol}–polyhedral volume; BLD-bond length distortion [22], TAV-tetrahedral angle variance [23], TQE-tetrahedral quadratic elongation [23].

Atom	ABL	P_{vol}	BLD	TQE/ OQE	TAV/ OAV	ABL	P_{vol}	BLD	TQE/ OQE	TAV/ OAV	Atom	ABL	P_{vol}	BLD	TQE/ OQE	TAV/ OAV	Atom
Zr-1	2.087	12.016	1.827	1.0064	19.9858	Si2-1	1.626	2.206	1.523	1.0010	3.0596	Si3-1	1.624	2.193	0.651	1.0022	8.3915
Zr-2	2.087	12.016	1.827	1.0064	19.9857	Si2-2	1.626	2.206	1.523	1.0010	3.0595	Si3-2	1.624	2.193	0.651	1.0022	8.3914
Zr-3	2.087	12.016	1.827	1.0064	19.9857	Si2-3	1.626	2.206	1.523	1.0010	3.0595	Si3-3	1.624	2.193	0.651	1.0022	8.3914
Zr-4	2.087	12.016	1.827	1.0064	19.9857	Si2-4	1.626	2.206	1.523	1.0010	3.0596	Si3-4	1.624	2.193	0.651	1.0022	8.3915
Si1-1	1.624	2.193	0.650	1.0022	8.3938	Si2-5	1.626	2.206	1.523	1.0010	3.0595	Si3-5	1.624	2.193	0.651	1.0022	8.3914
Si1-2	1.624	2.193	0.650	1.0022	8.3938	Si2-6	1.626	2.206	1.523	1.0010	3.0597	Si3-6	1.624	2.193	0.651	1.0022	8.3915
Si1-3	1.624	2.193	0.650	1.0022	8.3939	Si2-7	1.626	2.206	1.523	1.0010	3.0597	Si3-7	1.624	2.193	0.651	1.022	8.3915
Si1-4	1.624	2.193	0.650	1.0022	8.3938	Si2-8	1.626	2.206	1.523	1.0010	3.0595	Si3-8	1.624	2.193	0.651	1.0022	8.3914
Si1-5	1.624	2.193	0.650	1.0022	8.3938							Ca2-1	2.396	16.972	1.302	1.0532	166.6241
Si1-6	1.624	2.193	0.650	1.0022	8.3938							Ca2-2	2.396	16.972	1.302	1.0532	166.6241
Si1-7	1.624	2.193	0.650	1.0022	8.3938							Ca2-3	2.396	16.972	1.302	1.0532	166.6238
Si1-8	1.624	2.193	0.650	1.0022	8.3938							Ca2-4	2.396	16.972	1.302	1.0532	166.6238

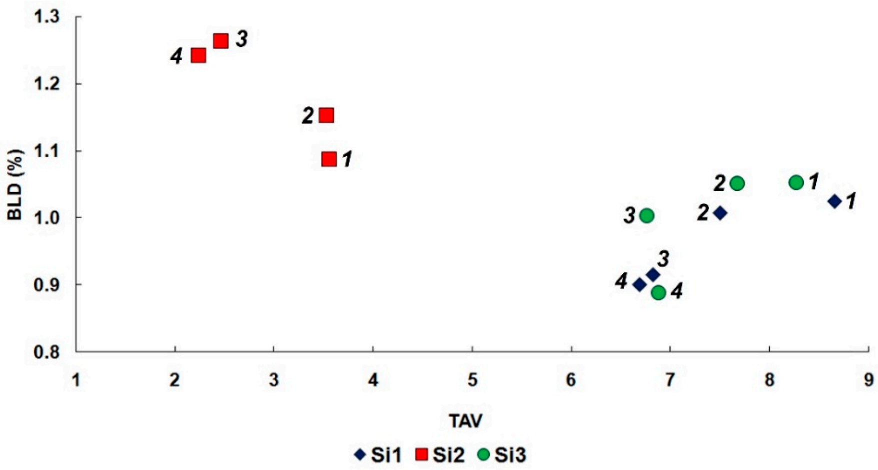

Figure 1. Bond length distortion (BLD) for tetrahedral of elpidite crystal structure against tetrahedral angle variance (TAV). 1 and 2 are ElB1 and ElB2–elpidite from Burpala (Russia); 3 and 4 are ElKhB1and ElKhB2–elpidite from Khan-Bogdo (Mongolia).

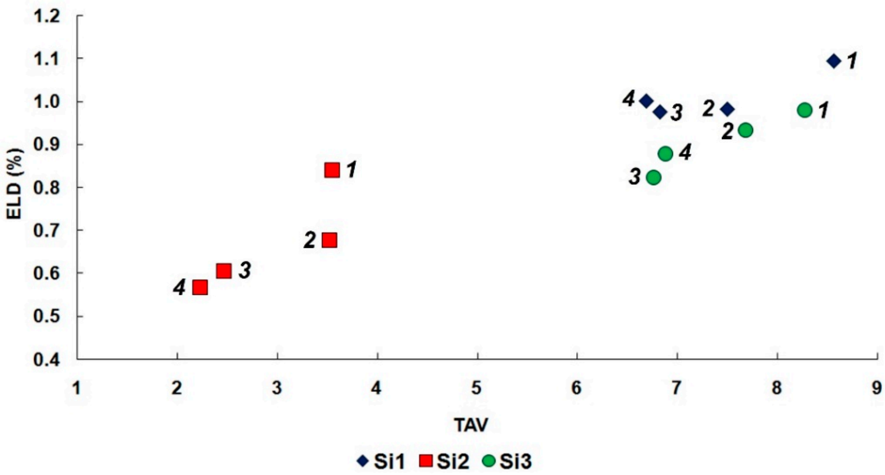

Figure 2. Edge length distortion (ELD) for tetrahedral of elpidite crystal structure against tetrahedral angle variance (TAV). 1 and 2 are ElB1 and ElB2–elpidite from Burpala (Russia); 3 and 4 are ElKhB1and ElKhB2–elpidite from Khan-Bogdo (Mongolia).

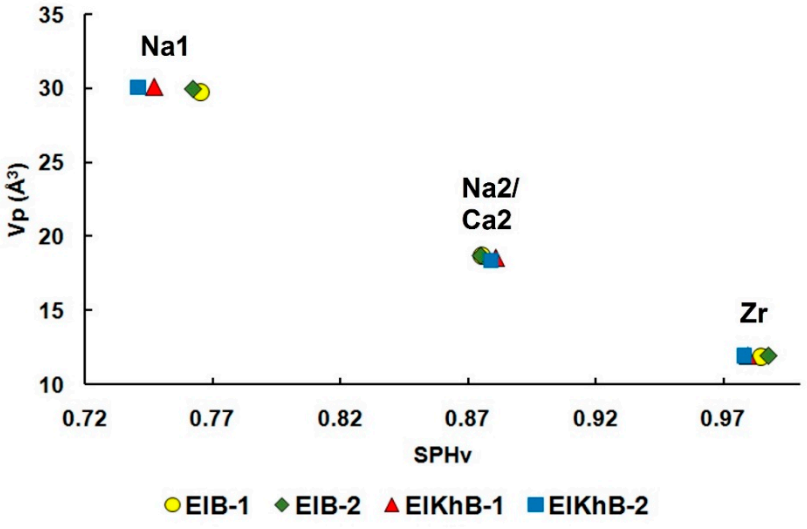

Figure 3. The volume of the coordination polyhedra (Vp) of elpidite crystal structure against polyhedra volume sphericity (SPHv).

References

1. Neronova, N.N.; Belov, N.V. Crystal structure of elpidite Na2ZrSi6O15·(H2O)3. Dimorphism of the dimetasili-cate radical Si6O15. Dokl. Akad. Nauk SSSR. 1963, 150, 642–645.
2. Neronova, N.N.; Belov, N.V. Crystal structure of elpidite, Na2ZrSi6O15· (H2O)3. Sov. Phys. Crystallogr. 1964, 9, 700–705.
3. Chao, G.Y. Leucophanite, elpidite, narsarsukite from the Desourdy quarry, Mont St. Hilaire, Quebec. Can. Min. 1967, 9, 286-287.
4. Cannillo, E.; Rossi, G.; Ungaretti, L. The crystal structure of elpidite. Am. Mineral. J. Earth Planet. Mater. 1973, 58, 106–109.
5. Sapozhnikov, A.N.; Kashaev, A.A. Features of the crystal structure of calcium-containing elpidite. Sov. Phys. Crystallogr. 1978, 23, 24–27.
6. Zubkova, N.V.; Ksenofontov, D.A.; Kabalov, Y.K.; Chukanov, N.V.; Nedel'ko, V.V. Dehydration-induced struc-tural transformations of the microporous zirconosilicate elpidite. Inorg. Mater. 2011, 47, 506–512. doi.org/10.1134/S0020168511050232.
7. Grigor'Eva, A.A.; Zubkova, N.V.; Pekov, I.V.; Kolitsch, U.; Pushcharovsky, D.Y.; Vigasina, M.F.; Giester, G.; Dordevic, T.; Tillmanns, E.; Chukanov, N.V. Crystal chemistry of elpidite from Khan Bogdo (Mongolia) and its K- and Rb-exchanged forms. *Crystallogr. Rep.* **2011**, *56*, 832–841, doi:10.1134/s1063774511050117.
8. Seryotkin, Y.V.; Bakakin, V.V.; Pekov, I.V. Structural evolution of microporous zirconosilicate elpidite under high pressure. *J. Struct. Chem.* **2014**, *55*, 1252–1259, doi:10.1134/s0022476614070087.
9. Grice, J.D.; Rowe, R.; Poirier, G. Hydroterskite: A New Mineral Species From the Saint-Amable Sill, Quebec, and A Comparison With Terskite and Elpidite. *Can. Miner.* **2015**, *53*, 821–832, doi:10.3749/canmin.1400105.
10. Cametti, G.; Armbruster, T.; Nagashima, M. Dehydration and thermal stability of elpidite: An in-situ single crystal X-ray diffraction study. *Microporous Mesoporous Mater.* **2016**, *227*, 81–87, doi:10.1016/j.micromeso.2016.02.049.
11. Zubkova, N.V.; Nikolova, R.P.; Chukanov, N.V.; Kostov-Kytin, V.V.; Pekov, I.V.; Varlamov, D.A.; Larikova, T.S.; Kazheva, O.N.; Chervonnaya, N.A.; Shilov, G.V.; et al. Crystal Chemistry and Properties of Elpidite and Its Ag-Exchanged Forms. *Miner.* **2019**, *9*, 420, doi:10.3390/min9070420.
12. Kostov-Kytin, V.V.; Kerestedjian, T.N. Rietveld Analysis of Elpidite Framework Flexibility Using in Situ Powder XRD Data of Thermally Treated Samples. *Miner.* **2020**, *10*, 639, doi:10.3390/min10070639.
13. Zubkova, N.V.; Pekov, I.V.; Chukanov, N.V.; Yapaskurt, V.O.; Turchkova, A.G.; Larikova, T.S.; Pushcharovsky, D.Y. A highly hydrated variety of elpidite from the Khibiny alkaline complex, Kola Peninsula, Russia. *Miner. Mag.* **2020**, 1–7, doi:10.1180/mgm.2020.96.
14. Gagné, O.C.; Hawthorne, F.C. Comprehensive derivation of bond-valence parameters for ion pairs involving oxygen. *Acta Crystallogr. Sect. B Struct. Sci. Cryst. Eng. Mater.* **2015**, *71*, 562–578, doi:10.1107/s2052520615016297.
15. 15. Hoppe, R. Effective coordination numbers (ECoN) and mean fictive ionic radii (MEFIR). Z. für Kristallogr. 1979, 150, 23–52. doi.org/10.1524/zkri.1979.150.14.23.
16. Hoppe, R.; Voigt, S.; Glaum, H.; Kissel, J.; Müller, H.P.; Bernet, K. A new route to charge distributions in ionic solids. *J. Less Common Met.* **1989**, *156*, 105–122, doi:10.1016/0022-5088(89)90411-6.
17. Nespolo, M.; Ferraris, G.; Ohashi, H. Charge distribution as a tool to investigate structural details: meaning and application to pyroxenes. *Acta Crystallogr. Sect. B Struct. Sci.* **1999**, *55*, 902–916, doi:10.1107/s0108768199008708.

18. Momma, K.; Izumi, F. VESTA 3for three-dimensional visualization of crystal, volumetric and morphology data. *J. Appl. Crystallogr.* **2011**, *44*, 1272–1276, doi:10.1107/s0021889811038970.
19. Žunić, T.B.; Makovicky, E. Determination of the centroid or `the best centre' of a coordination polyhedron. *Acta Crystallogr. Sect. B Struct. Sci.* **1996**, *52*, 78–81, doi:10.1107/s0108768195008251.
20. Balić-Žunić, T.; Vicković, I. IVTON – program for the calculation of geometrical aspects of crystal structures and some crystal chemical applications. J. Appl. Cryst. 1996, 29, 305–306. doi.org/10.1107/S0021889895015081.
21. Makovicky, E.; Balić-Žunić, T. New measure of distortion for coordination polyhedra. Acta Crystallogr. 1998, B54, 766–773. doi.org/10.1107/S0108768198003905.
22. Renner, B.; Lehmann, G. Correlation of angular and bond length dis¬tortions in TO4 units in crystals. Z. für Kris-tallogr. 1986, 175, 43–59 (1986). doi.org/10.1524/zkri.1986.175.1-2.43.
23. Robinson, K.; Gibbs, G.V.; Ribbe, P.H. Quadratic elongation: A quantita¬tive measure of distortion in coordina-tion polyhedra. Science. 1971, 172, 567–570. doi.org/10.1126/science.172.3983.567.
24. Chukanov, N.V. Infrared spectra of mineral scpecies. Springer, Dordrecht, 2014. doi.org/10.1007/978-94-007-7128-4.
